# Scanning Tunnelling Microscopy Study of Spin Interactions in Transition‐Metal Phthalocyanine Adsorbates: Mechanisms, Signatures, and Control Strategies

**DOI:** 10.1002/advs.202600003

**Published:** 2026-04-20

**Authors:** Fudi Zhou, Can Zhang, Zhaoteng Dong, Mengya Ren, Lili Zhou, Yu Zhang, Yeliang Wang

**Affiliations:** ^1^ School of Integrated Circuits and Electronics School of Interdisciplinary Science MIIT Key Laboratory For Low‐Dimensional Quantum Structure and Devices Beijing Institute of Technology Beijing China; ^2^ Key Laboratory of Multiscale Spin Physics(Ministry of Education) Beijing Normal University Beijing China

**Keywords:** Kondo effect, spin interactions, scanning tunnelling microscopy, transition‐metal phthalocyanine molecules

## Abstract

Spin interactions between magnetic molecules and substrates are of fundamental importance, as they offer key insights into spin‐related phenomena and underpin the development of energy‐efficient spintronic devices. Transition‐metal phthalocyanine (TMPc) molecules serve as exemplary model systems for probing these interactions, owing to their high structural stability, tunable magnetic moments, and well‐defined local environment. When adsorbed on different substrates, TMPc molecules can exhibit a rich variety of spin‐related phenomena including the Kondo effect, spin excitations, and Yu–Shiba–Rusinov (YSR) states, which can be directly resolved by scanning tunnelling microscopy (STM). This review summarizes recent STM advances on the coexistence and competition of spin‐related phenomena in TMPc adsorbates, emphasizing the underlying mechanisms and practical control strategies enabled by chemical design and electric‐ and magnetic‐field stimuli, and concludes with an outlook on emerging directions for future research.

## Graphical abstract

Transition‐metal phthalocyanine (TMPc) molecules serve as model systems for probing the spin interactions. This review summarizes recent scanning tunnelling microscopy advances on the spin‐related phenomena in TMPc adsorbates, including Kondo effect, spin excitations, and Yu–Shiba–Rusinov states, emphasizing the mechanisms and control strategies, and concludes the emerging directions for future research.

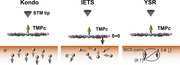

## 1 Introduction

Spintronics is a rapidly developing frontier in condensed matter physics and nanoscience. It aims to utilize the electron spin degree of freedom, together with its coupling with charge and orbital degrees of freedom, to achieve efficient information transport and storage [1, 2, 3, 4, 5]. Compared with conventional electronics that rely primarily on charge transport, spintronic systems offer compelling advantages in energy efficiency, operating speed, and device miniaturization. Moreover, they provide a versatile platform for exploring quantum computing, topological quantum states, and many‐body quantum phenomena [6, 7, 8, 9, 10, 11, 12, 13, 14]. In this framework, spin interactions at the atomic and molecular scales are a fundamental prerequisite for efficient spin manipulation and readout [15]. The strength and nature of these interactions directly determine the characteristics of spin interactions and the efficiency of spin information transport [16, 17, 18, 19]. Therefore, achieving precise and tunable control of spin interaction is of great significance to the development of spintronic technologies.

Among the various materials used to realize molecular spin functionalities, TMN_4_ coordination systems represent an important and widely studied class of platforms, in which a transition‐metal center is coordinated by four nitrogen atoms. Such motifs are commonly found in porphyrins, phthalocyanines, and related coordination complexes. In these systems, the local spin state is determined by the interplay of crystal‐field splitting, orbital hybridization, and electron correlation. It can be further tuned by the choice of metal center, the coordination environment, and the coupling to the local surroundings. Within this class, transition‐metal phthalocyanine (TMPc) molecules are particularly attractive model systems. Their rigid and well‐defined planar geometry ensures high structural uniformity. The extended π‐conjugated framework also provides excellent chemical and electronic stability, together with reproducible coupling to substrates. In addition, the central metal ion can be readily varied, allowing systematic tuning of the *d*‐orbital configuration and accessible spin states [20, 21, 22, 23, 24].

**FIGURE 1:**
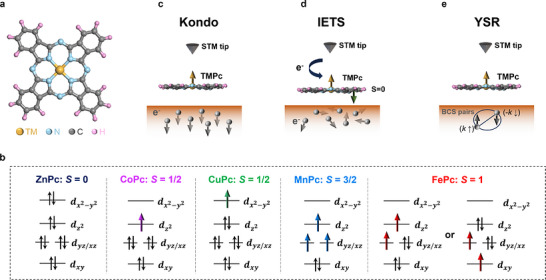
Structural and electronic properties of TMPc molecules. (a) Atomic model of an individual TMPc molecule. (b) The *d*‐orbital occupations and corresponding spin states of several gas‐phase TMPc molecules. For FePc, we show two possible spin configurations (having similar energy). (c–e) Schematic illustration of the principles of the Kondo effect, spin‐flip IETS, and YSR states.

For these reasons, TMPc molecules have emerged as prototypical systems for investigating molecular spin physics and spintronic properties. Structurally, a TMPc molecule comprises a central transition‐metal ion coordinated by a large π‐conjugated phthalocyanine macrocycle, yielding an approximately D_4h_ symmetric geometry (Figure 1a) [25, 26, 27, 28]. Within the ligand field of the phthalocyanine ring, the *d*‐orbitals of the central metal ion split into distinct energy levels. The resulting energy ordering and electron occupancy ultimately determine the total magnetic moment and spin configuration of the molecule.

The representative *d*‐orbital occupations and corresponding spin states of several gas‐phase TMPc molecules intuitively illustrate how variations in the electronic configuration of the central metal give rise to distinct magnetic properties. As summarized in Figure 1b, ZnPc features a fully filled *d* shell and thus adopts a closed‐shell configuration with spin *S* = 0. CoPc and CuPc both exhibit *S* = 1/2, yet the unpaired electron originates from different orbitals: in CoPc, it primarily occupies the $d_{z^{2}}$ orbital, whereas in CuPc, it resides in the $d_{x^{2}-y^{2}}$ orbital. MnPc displays *S* = 3/2, arising from three unpaired electrons distributed among the $d_{z^{2}}$ and *d*_*yz*/*xz*_ orbitals. FePc exhibits *S* = 1 and can adopt two possible electronic configurations: in one, the unpaired electrons occupy $d_{z^{2}}$ and *d*_*yz*/*xz*_, in the other, they are located in *d_xy_* and *d*_*yz*/*xz*_ orbitals. Consequently, by selecting different transition‐metal centers, the spin quantum number, orbital degeneracy, and magnetic anisotropy can be systematically tuned, thereby enabling precise control of local magnetic moments and tailored spin‐coupling characteristics at the molecular scale. This high degree of tunability makes TMPc molecules an ideal platform for elucidating the intrinsic interactions between electronic and magnetic properties at the single‐molecule level [29, 30, 31].

When adsorbed on different substrates, TMPc molecules can exhibit a rich variety of spin‐related phenomena. The interactions between the molecules and the substrates promote hybridization between the *d*‐orbitals of the central metal ion and the electronic states of the substrate, often accompanied by charge transfer, potential scattering, and related interfacial processes. As a result, diverse quantum phenomena may arise depending on the local environments, including Kondo resonances (Figure 1c) [32, 33, 34], inelastic electron tunneling spectroscopy (IETS) signatures (Figure 1d) [35, 36, 37], and Yu–Shiba–Rusinov (YSR) states (Figure 1e) [38]. By tuning the molecular composition, adsorption configuration, molecule–substrate coupling strength, and intermolecular arrangement, these quantum responses can be continuously and reversibly controlled over a broad parameter space. This high degree of tunability enables systematic interrogation of the subtle competition and interplay among local spins, conduction electrons, lattice vibrations, and superconducting pairing interactions [39, 40, 41, 42, 43, 44, 45, 46]. Therefore, TMPc‐based systems provide versatile model platforms for systematically investigating spin–electron correlations, quantum‐state engineering, and many‐body interactions at the molecular scale.

Low‐temperature scanning tunnelling microscopy and spectroscopy (STM/STS) play a pivotal role in exploring the electronic properties of surface‐adsorbed molecular systems, enabled by their atomic‐scale spatial resolution and millielectronvolt energy resolution. STM enables direct visualization of the molecular morphology, adsorption configuration, and intermolecular arrangement of TMPc molecules on various surfaces, whereas STS provides information access to the local density of states (LDOS) [47, 48, 49, 50, 51, 52]. Together, these techniques allow the identification of molecular orbital hybridization, spin excitations, Kondo resonances, and YSR states, thereby elucidating the interplay between electronic and spin degrees of freedom at molecule–substrate interfaces.

Computational approaches, particularly first‐principles calculations based on density functional theory (DFT), provide an important theoretical framework for analyzing spin interactions at molecule–substrate interfaces. For TMPc molecules adsorbed on surfaces, DFT calculations can determine the adsorption geometry, spin polarization, and magnetic moment of the transition‐metal center. This enables analysis of how molecule–substrate coupling modifies the molecular spin state. These methods also allow quantitative investigation of interfacial mechanisms governing spin coupling, including hybridization between molecular orbitals and substrate electronic states, charge transfer across the interface, and exchange interactions mediated by substrate electrons. In combination with STM and spectroscopy, such calculations facilitate the interpretation of experimentally observed spin‐related phenomena. The inclusion of spin–orbit interaction (SOI) additionally allows evaluation of magnetic anisotropy and related spin‐dependent properties. Together, STM measurements and theoretical modeling provide a consistent microscopic description of spin interactions in TMPc–substrate systems.

In this review, we summarize recent advances in the study of spin interactions between TMPc molecules and diverse substrates. We highlight representative studies on spin‐state modulation achieved through tuning the chemical composition, adsorption geometry, and external stimuli, and discuss the current challenges as well as future prospects for this rapidly evolving research field.

## 2 Spin Interactions of TMPc Molecules on Different Substrates

### 2.1 Kondo Effect of TMPc Adsorbates

The Kondo effect originates from the exchange interaction between the localized spin of a magnetic impurity and the conduction electrons in a metallic host. When the temperature is below the characteristic Kondo temperature, this interaction gives rise to coherent spin‐flip scattering, a many‐body process that effectively screens the localized magnetic moment. In STS spectra, the Kondo effect manifests as a pronounced resonance near the Fermi level, commonly referred to as the Kondo resonance [53, 54, 55, 56]. In recent years, STM/STS has been widely employed to explore Kondo physics in TMPc molecules adsorbed on a variety of substrates [32, 33, 34, 57]. These investigations have provided crucial experimental insights into spin–electron interactions at the molecular scale. Nevertheless, in many molecule–substrate systems, substantial charge transfer and strong interfacial chemical bonding can partially or completely quench the localized magnetic moment, thereby suppressing the emergence of a characteristic Kondo resonance at low temperatures [58, 59, 60, 61, 62]. Consequently, the rational selection and engineering of substrates are essential for realizing and controlling molecular Kondo effects. An appropriate balance between electronic coupling strength and chemical inertness is required.

Here, we first review STM studies of the Kondo effect in an individual TMPc molecule adsorbed on a substrate [33]. And then we discuss how Ruderman–Kittel–Kasuya–Yosida (RKKY) interactions between adjacent molecules can modify the Kondo response, and highlight high‐spatial‐resolution probing of molecular Kondo phenomena using spin‐polarized STM (SP‐STM) tips [32, 34].

**FIGURE 2:**
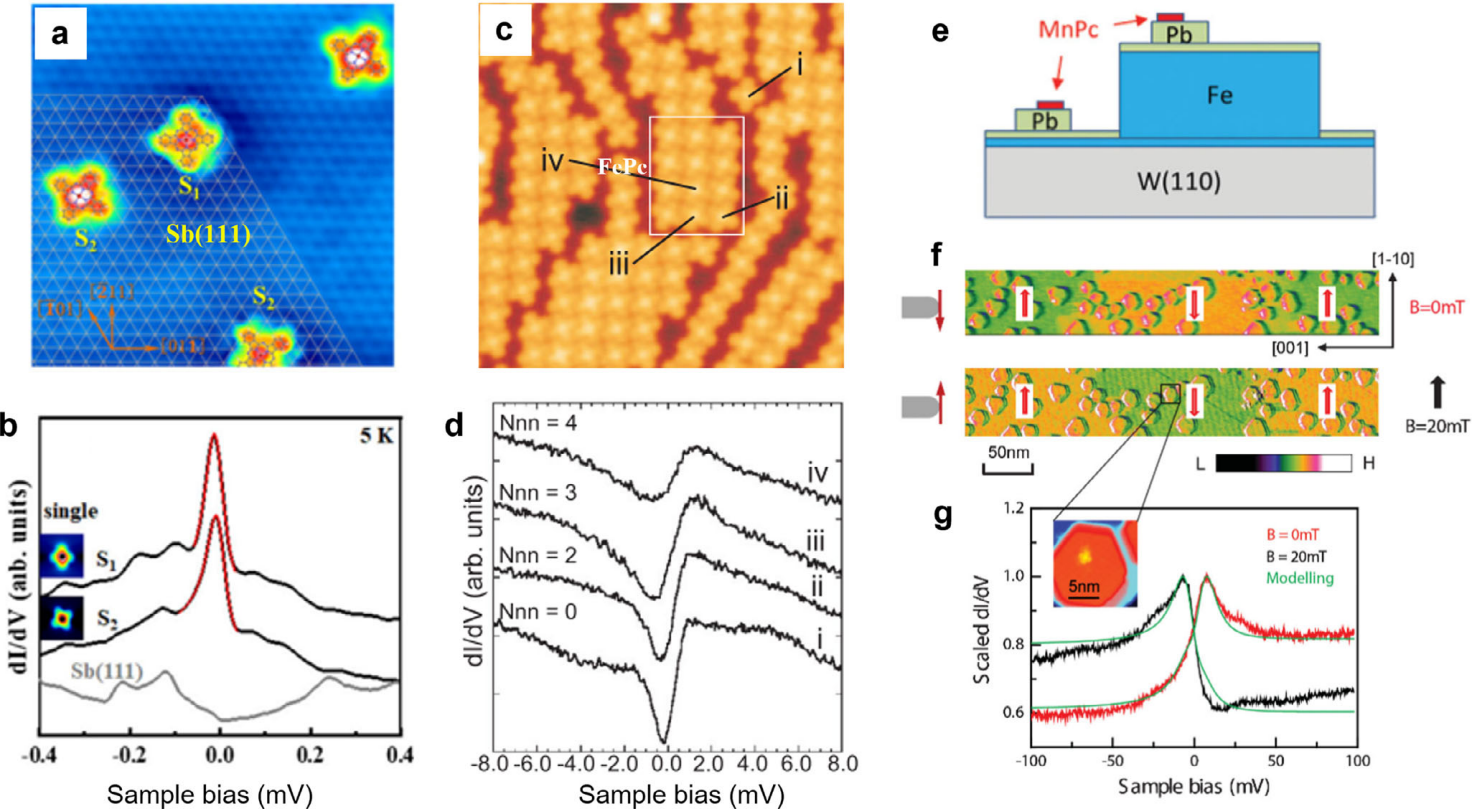
Kondo effect of TMPc adsorbates. (a) STM image of CoPc molecules on Sb(111) substrate (9.1 × 9.1 nm^2^, *I* = 300 pA, and *U* = 0.4 V), showing two configurations (S_1_ and S_2_), where the molecules are adsorbed at the bridge site of the substrate. (b) *dI/dV* spectra acquired at the central Co ions of the isolated CoPc molecules on the Sb(111) surface at 5 K. Adapted with permission [33]. Copyright 2022, American Physical Society. (c) Large‐scale STM image of FePc molecules on Au(111) surface (20 × 20 nm^2^, *I* = 100 pA, and *U* = ‐100 mV). (d) *dI/dV* spectra acquired on the FePc molecules at the locations marked in panel c at 0.4 K. Adapted with permission. [32] Copyright 2011, American Physical Society. (e) Schematic illustration of MnPc molecules adsorbed on Pb islands, which are grown on Fe nanostripes supported by a W(110) substrate. (f) Spin‐polarized *dI/dV* maps of a Pb‐decorated Fe nanostripe acquired at 0.5 V, revealing a clear magnetic contrast. The magnetization directions of the tip and the sample domains are schematically indicated. (g) Spin‐polarized *dI/dV* spectra of MnPc measured with a Fe‐coated W tip, showing a spin‐split Kondo resonance. Adapted with permission [34]. Copyright 2012, American Physical Society.

On the semimetallic Sb(111) surface, the coupling between CoPc molecules and the substrate is relatively weak, allowing the molecular magnetic moment to remain well preserved [33]. This makes the CoPc/Sb(111) interface an ideal platform for investigating the Kondo effect. Due to the differences in adsorption orientation, CoPc molecules exhibit two distinct configurations on Sb(111) (Figure 2a). Pronounced Kondo resonances are observed in both configurations (Figure 2b), further confirming that the molecular spin of CoPc is well maintained on the Sb(111) surface. DFT calculations indicate that the Co–substrate distance and the strength of interfacial hybridization are primarily governed by the substrate density of states (DOS) near the Fermi level, which plays a crucial role in stabilizing the molecular spin and driving the CoPc–substrate hybrid system into the Kondo regime.

The Kondo effect screens the local magnetic moment through spin‐electron many‐body scattering, driving the system toward a nonmagnetic ground state. In contrast, the RKKY coupling favors magnetic ordering between neighboring spins. The competition between these two interactions ultimately determines the magnetic ground state of the systems and gives rise to pronounced variations in the Kondo resonance. More specifically, the Kondo effect is associated with the screening of an individual localized spin and gives rise to a sharp resonance near the Fermi level. By contrast, the RKKY interaction couples nearby spins, reshapes their correlated spin state, and thereby affects the evolution of the Kondo resonance. Depending on the sign and strength of the RKKY coupling, as well as the coupling to the substrate, the Kondo resonance may evolve in different ways, including changes in intensity, width, or splitting [56, 63, 64, 65, 66]. Figure 2c shows FePc molecules forming two‐dimensional (2D) clusters on Au(111), where *N_nn_* represents the number of nearest‐neighbor (NN) molecules. Since the NN separation is ∼1.47 nm, both direct exchange and dipolar couplings are negligible. Thus, only the intermolecular RKKY interaction is considered [32]. The RKKY coupling is antiferromagnetic for NN pairs at ∼1.47 nm, ferromagnetic for second‐nearest neighbors at ∼2.07 nm, and weakly antiferromagnetic for third‐nearest neighbors at ∼2.94 nm. Among these contributions, the NN interaction (1.47 nm) dominates the overall spin coupling.

Within the theoretical framework of the two‐impurity Kondo problem, an antiferromagnetic RKKY coupling results in a broadening, suppression, and even splitting of the Kondo resonance, whereas a ferromagnetic RKKY coupling leads to a narrower and enhanced resonance [63, 64, 65]. Consequently, as the number of adjacent molecules increases, the effective antiferromagnetic coupling strengthens. This leads to a systematic spectral evolution. Molecules at the cluster corners, which have two adjacent molecules, exhibit spectra nearly identical to those of an isolated molecule (i). In contrast, the Kondo dip progressively broadens and becomes shallower as *N_nn_* increases from two (ii) to three (iii) and four (iv) (Figure 2d).

The studies discussed above on the Kondo effect in TMPc molecules were all performed using non–spin‐polarized STM. In contrast to a conventional non‐spin‐polarized STM tip, a spin‐polarized STM tip enables the detection of spin‐resolved tunneling signals and thereby disentangle contributions from different spin channels. This capability makes it possible to directly reveal the spin‐polarized characteristics of the Kondo state and its spin‐dependent splitting behavior [67, 68, 69, 70, 71]. As shown in Figure 2e, the ratio of the interlayer spacing and the Fermi wavelength of Pb(111) is about 1:4. According to $J=J_{0}\;\frac{cos(2k_{F}d)}{{\left(2k_{F}d\right)}^{D}}$[72], an antiferromagnetic RKKY interaction is expected between the MnPc molecule on Pb and the underlying magnetic Fe substrate, lifting the original twofold spin degeneracy. Here, *J* is the indirect exchange coupling strength, *J_0_* is a prefactor related to the coupling constant, *k_F_* is the Fermi wave vector, *d* is the separation between the two magnetic moments, and *D* is the dimensionality of the conduction electrons. Using a spin‐averaged bare W tip, a spin‐split Kondo resonance is observed on the MnPc molecule.

Further spin‐polarized STM measurements with a Fe‐coated W tip, whose magnetic sensitivity is verified by the clear contrast on a Pb‐decorated Fe nanostripe (Figure 2f), reveal a pronounced Kondo resonance of MnPc that shifts from +7.1 mV at zero magnetic field to −6.3 mV at 20 mT (Figure 2g) [68]. Assuming that the DOS of the Fe‐coated tip near the Fermi level is dominated by majority spin states, the spin character of the split resonances can thus be identified: the occupied resonance corresponds to the minority spin, while the unoccupied resonance corresponds to the majority spin [73]. These observations provide further evidence that the RKKY coupling between MnPc and the Fe substrate is antiferromagnetic. Therefore, the spin‐dependent splitting of the Kondo resonance provides direct confirmation of the spin‐polarized nature of the RKKY interaction and the antiferromagnetic coupling between the MnPc molecules and the Fe substrate [34].

Overall, the Kondo effect in TMPc molecules depends on the preservation of the molecular localized spin after adsorption and on the establishment of exchange coupling with the substrate conduction electrons. The relative alignment of molecular orbitals with respect to the substrate Fermi level is a key factor. The strength of molecule–substrate hybridization, interfacial charge transfer, and the local coordination environment also influence the stability of the magnetic moment and the formation of the Kondo resonance. In addition, indirect exchange interactions between neighboring molecules can further modulate the spectral response. Control of the substrate electronic properties and the interfacial structure, therefore, provides an effective route to realizing and tuning molecular Kondo effects.

### 2.2 Spin‐Flip IETS of TMPc Adsorbates

Spin‐flip IETS is a highly sensitive STM‐based technique for probing spin excitation processes at the atomic and molecular scales. When the energy of a tunneling electron exceeds the excitation threshold of a localized spin system, a fraction of its energy and angular momentum can be transferred to the molecular spin via an inelastic tunneling channel, thereby inducing transitions between spin states. In differential conductance (*dI/dV*) spectra, such inelastic processes appear as symmetric step‐like features at both positive and negative bias. Correspondingly, the second derivative (*d^2^I/dV^2^*) spectra exhibit peaks, with the energies directly corresponding to the spin excitation energies [74, 75, 76, 77, 78, 79].

For magnetic TMPc molecules, the partially filled *d*‐orbitals of the central metal ion act as a localized spin center, while the ligand field, together with SOI, determines the molecular magnetic anisotropy and spin multiplicity. Spin‐flip IETS enables quantitative determination of the spin excitation level scheme, magnetic anisotropy parameters, and the transition pathways between spin states in TMPc molecules [35, 36, 37, 80, 81, 82, 83, 84]. Moreover, changing the central metal ions can produce distinct spin‐excitation fingerprints. This provides key experimental insights into the underlying electronic structure, coordination environment, and magnetic tuning mechanisms of transition‐metal centers in these molecular systems.

Here, we mainly focus on three representative categories of TMPc molecular systems investigated using spin‐flip IETS. The first addresses spin excitations and magnetic anisotropy in a single molecule, where IETS resolves the splitting of the spin energy levels of the central metal ion and enables quantitative extraction of magnetic anisotropy parameters, thereby clarifying the microscopic origin of single‐molecule magnetism [35]. The second concerns singlet–triplet transitions within individual molecules, in which IETS captures the changes in spin states induced by variations in coordination environment or charge state, highlighting the tunability and quantum nature of molecular spin multiplicity [36]. The third focuses on molecular chains and assemblies, in which IETS directly tracks how spin excitations evolve under intermolecular exchange or RKKY‐mediated interactions, providing insight into collective magnetic behavior in coupled molecular systems [37].

Zero‐field splitting (ZFS) refers to the lifting of the degeneracy of spin sublevels in a system with total spin quantum number *S *≥ 1 in the absence of an external magnetic field. In such systems, states that would otherwise be degenerate with respect to the spin magnetic quantum number *m_S_* become split, due to the intrinsic magnetic anisotropy of the system [85]. This anisotropy arises predominantly from the second‐order spin–orbit interaction in a low‐symmetry crystal field, and it may be accompanied by weaker contributions from spin–spin dipolar interactions [86, 87, 88, 89, 90].

**FIGURE 3:**
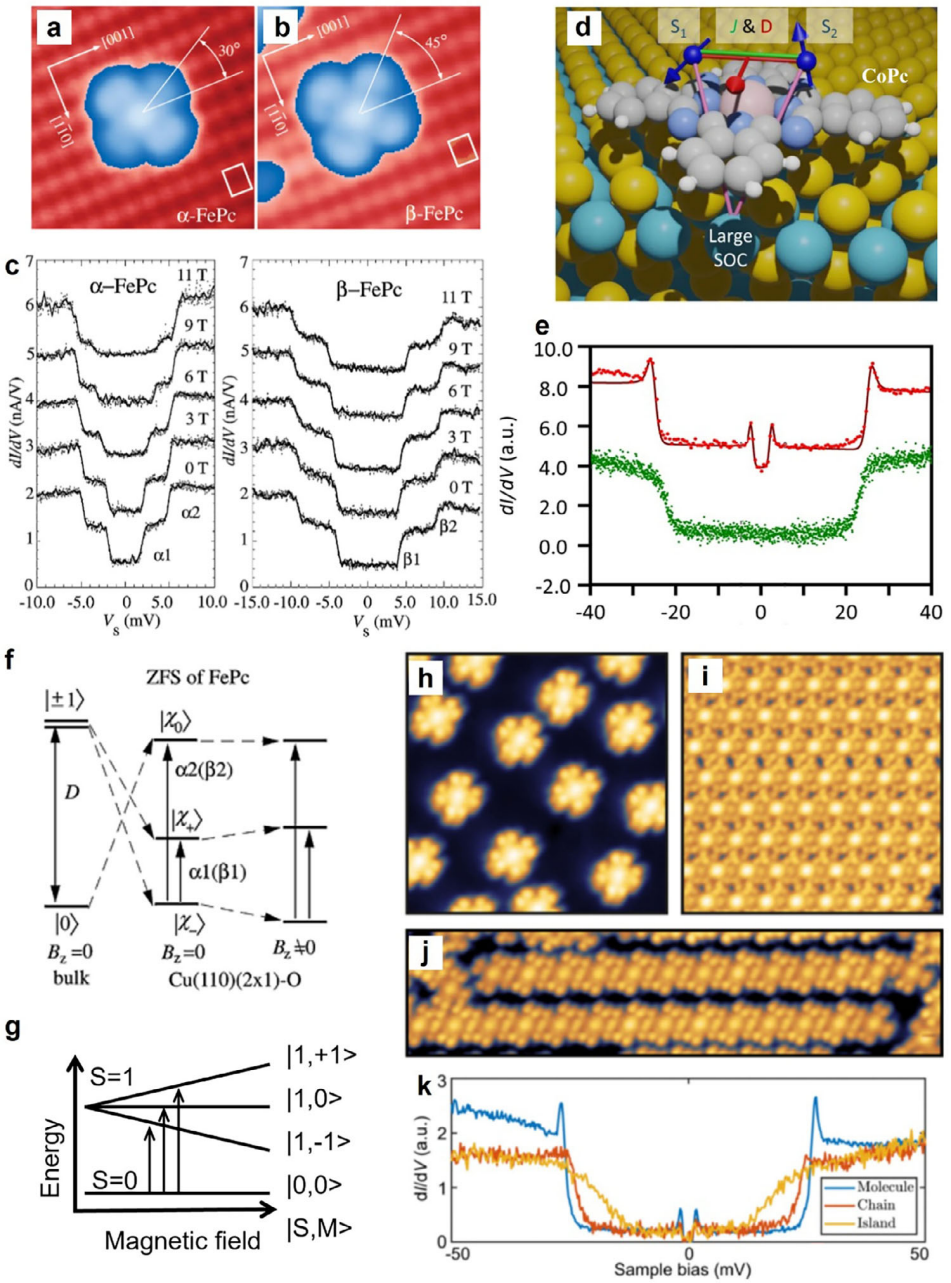
Spin‐flip IETS of TMPc adsorbates. (a,b) STM images of α‐ and β‐FePc on Cu(110)‐(2×1)‐O substrates taken at 0.4 K (4 **×** 4 nm^2^, *I* = 100 pA, and *U* = ‐0.5 V). (c) *dI/dV* spectra of FePc molecules on Cu(110)‐(2×1)‐O substrates in the two configurations at 0.4 K. Adapted with permission [35]. Copyright 2009, American Physical Society. (d) Schematic model of the singlet state CoPc molecule with *S* = 0, where the arrows represent the spin directions. (e) *dI/dV* spectra measured on CoPc at *B* = 0 (red dots) and at *B* = 8 T (green dots). The magnetic field *B* is perpendicular to the sample surface. Adapted under the terms of the Creative Commons CC BY license [36]. Copyright 2021, The Author(s). (f) The energy diagram of ZFS for bulk FePc and a single FePc molecule on Cu(110)‐(2×1)‐O. *D* denotes the axial zero‐field splitting parameter. ∣0〉 and ∣ ± 1〉 represent the spin triplet states in the bulk, where ∣ ± 1〉 are degenerate at *B_z_* =  0. On the surface, transverse anisotropy lifts this degeneracy and mixes the ∣ ± 1〉 states, resulting in new eigenstates ∣χ_0_〉, ∣χ_+_〉, and ∣χ_−_〉. α_*i*_and β_*i*_ denote the corresponding mixing coefficients. *B*_*z* _is the external magnetic field along the z direction. Adapted with permission [35]. Copyright 2009, American Physical Society. (g) Energy‐level diagram of spin excitation from the singlet (*S*  =  0) to triplet (*S*  =  1) state of CoPc on bulk NbSe_2_ under an external magnetic field. *S*denotes the total spin, and ∣*S*, *M*〉 represents the spin eigenstates with magnetic quantum number *M*. The degeneracy of the triplet sublevels is lifted by the magnetic field. (h) CoPc adsorbs on NbSe_2_ at a low coverage, where CoPc can be regarded as individual molecules. (i,j) The CoPc molecules self‐assemble into molecular islands and chains as the coverage increases. (k) *dI/dV* spectra acquired above the center atom of an individual CoPC (blue), a molecule in a chain (orange), and one in an island (yellow) at 4 K. The steplike features in the spectra correspond to spin excitations of the molecule by inelastic tunneling. Adapted with permission [37]. Copyright 2023, American Physical Society.

When a single FePc molecule is adsorbed on the Cu(110)(2 × 1)‐O surface, two distinct adsorption configurations (α‐FePc and β‐FePc) are observed (Figure 3a,b). The molecular axes of these two configurations are rotated by approximately 30° and 45°, respectively, relative to the [001] direction of the substrate, reflecting different adsorption geometries within the surface troughs. At zero magnetic field, the *dI/dV* spectra of both α‐ and β‐FePc exhibit symmetric double‐step features with respect to zero bias (Figure 3c), characteristic of inelastic tunneling processes associated with spin‐flip excitations. These steps originate from ZFS within a triplet spin state (Figure 3f), induced by spin–orbit interaction at the Fe center in conjunction with an anisotropic ligand field. The ZFS of FePc can be captured by the effective spin Hamiltonian,

$$\;{\hat{\;H}}_{\mathrm{eff}}=D\hat{S_{z}^{2}}+E\left(\hat{S_{x}^{2}}\;-\;\hat{S_{y}^{2}}\right),$$

where *D* is the axial ZFS constant that determines the magnetic anisotropy, and *E* represents the rhombicity of the molecule. For *D* > 0, the molecule exhibits easy‐plane magnetic anisotropy, whereas *D* < 0 corresponds to easy‐axis anisotropy.

Although α‐FePc and β‐FePc differ in adsorption geometries and anisotropy parameters, both display clear IETS steps arising from magnetic‐anisotropy‐driven splitting of the spin levels. Specifically, the two adsorption configurations exhibit distinct ZFS parameters. For α‐FePc, *D *= −3.8 meV and *E* = 1 meV, whereas for β‐FePc, *D *= −6.9 meV and *E* = 2.1 meV. The negative values of *D* indicate that adsorption on the Cu(110)(2×1)‐O surface switches the magnetic anisotropy of FePc from the easy‐plane character observed in the bulk to an easy‐axis anisotropy. Meanwhile, the nonzero *E* values reflect the reduction of ligand‐field symmetry caused by the anisotropic surface environment. As a result, the α/β polymorphism modifies the ligand‐field symmetry and magnetic anisotropy at the Fe center, leading to distinct spin‐excitation energies observed in IETS [35].

In contrast to systems with pronounced magnetic anisotropy, where spin excitations arise from ZFS induced by spin–orbit interaction, some systems without detectable anisotropy can still display pronounced spin‐related signatures in IETS [36, 81, 83, 161]. A representative example is CoPc molecules adsorbed on bulk 2H‐NbSe_2_: although no measurable magnetic anisotropy is found, distinct low‐temperature IETS features appear in the *dI/dV* spectra [36, 91]. This behavior originates from anti‐aligned spin polarizations on the central Co ion and the molecular lobes, yielding an overall singlet ground state (*S* = 0). The observed IETS features, therefore, correspond to singlet‐to‐triplet excitations (Figure 3d,e) [83]. The schematic illustration of the singlet‐to‐triplet transition and excitation is presented in Figure 3g. These results indicate that, even in the absence of magnetic anisotropy, intramolecular spin coupling can still give rise to spin‐flip processes that are accessible by IETS.

Beyond the localized spin excitations of isolated molecules, assemblies of CoPc molecules self‐assembled into chains and islands on NbSe_2_ exhibit collective spin excitations (Figure 3h–j) [37]. As intermolecular exchange interactions develop, the initially local singlet–triplet excitations become coupled and delocalized across neighboring molecules, giving rise to dispersive triplon excitations [92, 93]. Experimentally, this crossover is reflected in the *dI/dV* spectra as a gradual broadening of the inelastic tunneling steps and a systematic shift of the excitation energies toward lower values (Figure 3k). These features are consistent with stronger intermolecular coupling and local environmental variations. Detailed analysis indicates that exchange interactions between adjacent molecules hybridize individual molecular spin excitations, leading to the formation of a dispersive triplon excitation band. The bandwidth of this band increases with the coupling strength. As the exchange interaction increases, the excitations become more delocalized. Meanwhile, spatial inhomogeneity in coupling pathways and magnitudes contributes to the observed spectral broadening. Intermolecular magnetic exchange, therefore, plays a key role in governing the evolution of the spin‐excitation spectrum from localized molecular excitations to collective triplon excitations.

### 2.3 YSR States at TMPc–Superconductor Interfaces

A localized magnetic moment coupled to a conventional (s‐wave) superconductor interacts with the superconducting quasiparticles via exchange coupling. This interaction breaks Cooper pairs and gives rise to a localized bound state within the superconducting energy gap, known as the YSR state [94, 95, 96]. The YSR state embodies the competition between magnetic scattering and the superconducting condensate: the local spin tends to exchange couple with the quasiparticles, producing a localized in‐gap quasiparticle state, whereas superconducting pairing favors a nonmagnetic condensate. For weak exchange coupling, the YSR level resides close to the gap edge, and the system remains a superconducting ground state. As the coupling increases, the YSR energy moves toward the Fermi level and can cross zero at a critical coupling, signaling a ground‐state transition from a superconductivity‐dominated screened singlet to a magnetism‐dominated unscreened doublet [97, 98, 99, 100]. This bound‐state evolution reveals the quantum interplay between localized moments and superconducting pairing, providing a powerful experimental handle on magnetic–superconducting coupling. In real space, YSR states are strongly localized around the local magnetic moment, although on low‐dimensional substrates, they may extend over several nanometers. Their wavefunctions and energies are highly sensitive to the local spin, exchange strength, potential scattering, and the electronic structure of the substrate [101]. Importantly, YSR states can be directly probed by STM/STS [38, 102, 103, 104, 105].

**FIGURE 4:**
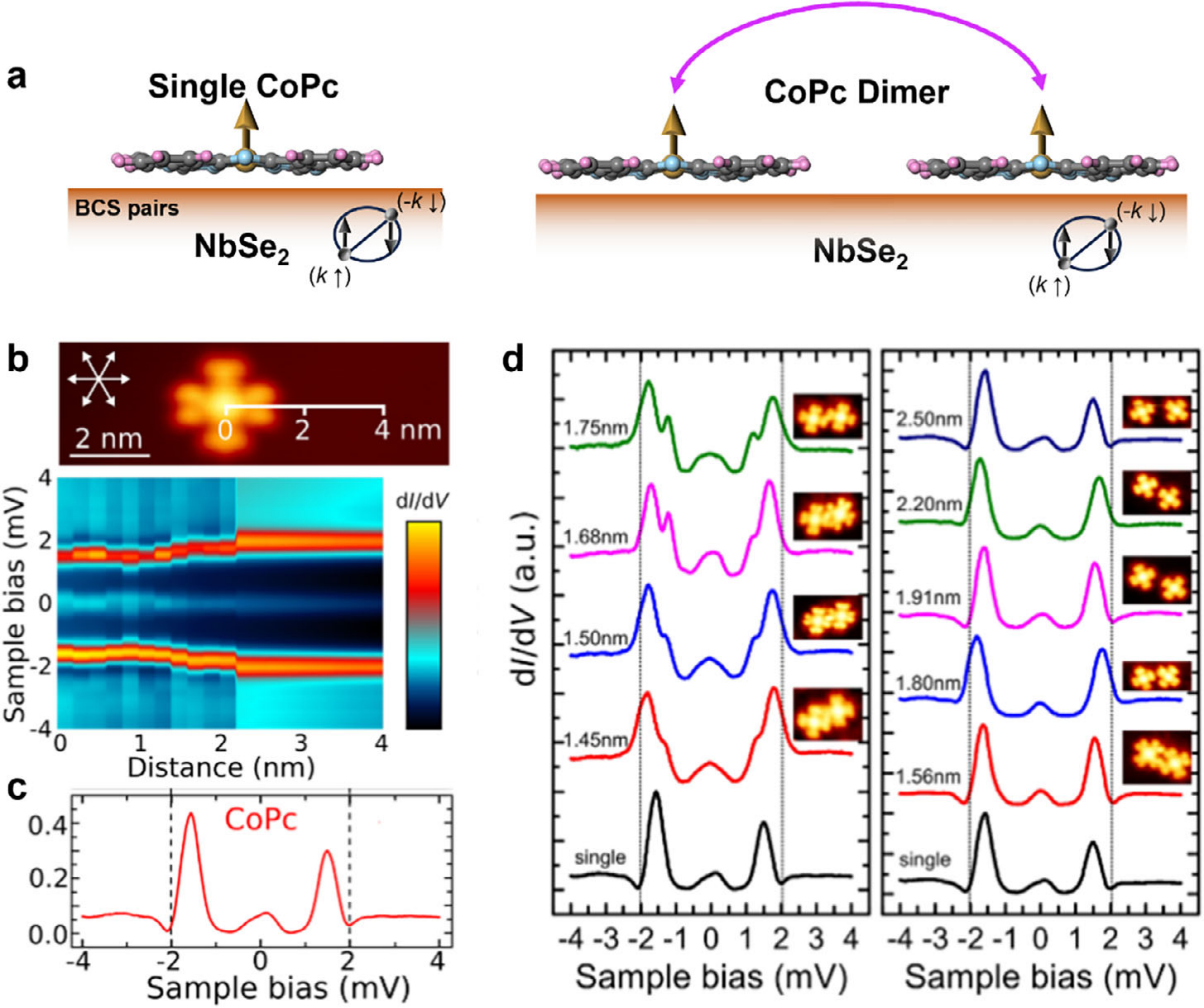
YSR states on an isolated CoPc molecule and CoPc dimer. (a) Schematic illustration of an isolated CoPc molecule and CoPc dimer adsorbed on the surface of superconducting NbSe_2_. (b) Spatially resolved *dI/dV* spectra, revealing the spatial evolution of the YSR resonances (feedback set point: *U* = 100 mV, *I* = 50 pA, *z_offset_* = 50 pm). Color scale between 0−1 µS. In the upper panel, the arrows indicate the principal directions of the underlying NbSe2 substrate. (c) *dI/dV* spectra recorded on an isolated CoPc molecule on NbSe_2_ substrates (feedback set point: *U* = 100 mV, *I* = 50 pA, *z_offset_* = 50 pm) with a superconducting STM tip. (d) A set of *dI/dV* spectra recorded on the CoPc dimer with distinct separations, showing split (left) and non‐split (right) YSR states. For comparison, a spectrum measured on an isolated CoPc molecule (black line) is shown in both panels (feedback set point: *U* = 100 mV, *I* = 50 pA, *z_offset_* = 100 pm). The dotted lines indicate the edge energies of the superconducting gap at ± 2Δ/e. Adapted under the terms of the Creative Commons CC BY license [38]. Copyright 2018, American Chemical Society.

TMPc molecules provide an excellent model system for investigating YSR physics. The central metal ion carries a localized spin that can establish a tunable exchange coupling to a superconducting substrate (Figure 4a). By adjusting the adsorption configuration and intermolecular arrangement, the molecule–substrate hybridization strength can be systematically modulated, enabling control of the YSR energy, particle–hole asymmetry, and real‐space spatial profile over a wide parameter range [38, 103, 106, 107]. This versatility makes TMPc–superconductor interfaces a powerful platform for exploring magnetic–superconducting interactions at the single‐molecule level and for constructing coupled YSR arrays.

NbSe_2_ is a prototypical layered transition‐metal dichalcogenide superconductor with a critical temperature of approximately 7.2 K. Its relatively long coherence length makes it well suited for investigating the interplay between localized magnetism and superconductivity. Compared with conventional three‐dimensional superconductors, the quasi‐2D electronic structure and reduced screening in NbSe_2_ can significantly enhance the spatial extent of impurity‐induced bound states [101, 108]. Consequently, the YSR wavefunctions may propagate over several nanometers along the surface lattice, facilitating coupling between nearby magnetic impurities.

STM/STS measurements of CoPc molecules adsorbed on NbSe_2_ reveal a pair of YSR resonances inside the superconducting gap (Figure 4b,c), originating from exchange coupling between the molecular spin and the superconducting host. When two CoPc molecules are positioned by STM manipulation to form a dimer, coupled YSR states emerge. In this case, the monomeric YSR pair splits into two distinct resonances, corresponding to the bonding and antibonding combinations of the hybridized YSR wavefunctions, with the splitting magnitude directly reflecting the intermolecular coupling strength. As the intermolecular spacing is reduced, the energy separation increases; when the molecules are separated by several nanometers, the spectra recover the single‐impurity YSR resonance (Figure 4d), indicating that the coupling is dominated by wavefunction overlap.

Theoretical analysis further shows that the interaction is mediated by the superconducting substrate, rather than by direct dipole coupling or an RKKY exchange between the molecular spins. This interpretation is further supported by the following fact. In two‐dimensional systems, RKKY interactions decay more rapidly with distance than YSR‐mediated coupling. Therefore, they are less important at the intermolecular separations considered here. In addition, alternative mechanisms such as dipolar coupling or orbital scattering would be expected to appear already at the single‐impurity level, where only a single pair of YSR states is observed.

On NbSe_2_, the YSR wavefunction decays exponentially but also oscillates in real space with a period determined by the Fermi wave vector *k_F_* [109, 110, 111]. This oscillatory behavior reflects the coherent propagation of superconducting quasiparticles in the substrate. The spatial period of the oscillation is set by the Fermi wave vector *k_F_*. As a result, the coupling between YSR states depends sensitively on intermolecular spacing and can display oscillatory behavior. This picture naturally accounts for the experimentally observed non‐monotonic dependence of the YSR splitting on molecular separation, and identifies substrate‐mediated coherent hybridization as the fundamental origin of molecular YSR coupling [38].

The crossover from a localized monomeric YSR state to a coherently hybridized intermolecular state highlights that, at molecule–superconductor interfaces, YSR states can be tuned not only through chemical or electronic control of the local spin states [112, 113] but also by precisely manipulating molecular arrangement and separations to continuously modulate the coupling strength. Such molecular‐scale engineering strategy enables the construction of YSR arrays with controllable coupling and emergent band structures, providing an experimental foundation for investigating magnetic–superconducting interactions, designing artificial many‐body bound states, and exploring precursor phases of topological superconductivity [111, 114, 115, 116].

### 2.4 Coexistence and Correlation Among Kondo, IETS, and YSR States

In many TMPc molecule–substrate systems, the Kondo effect, spin‐flip IETS signatures, and YSR states are generally not independent. Instead, their emergence and spectral characteristics are all governed by the exchange coupling between molecular spin and the conduction electrons from the substrate, as well as by the interplay with Cooper pairs in superconducting hosts. Although their microscopic mechanisms differ, their coexistence and competition are largely governed by the relative hierarchy of relevant energy scales, including the exchange coupling strength, magnetic anisotropy, the Kondo screening scale, and the superconducting gap.

The balance among these energy scales determines which interaction channel dominates the spectral response, while others may remain as subleading features. As these parameters vary, the system can evolve continuously between different regimes, enabling the coexistence, competition, or even hybridization of these many‐body states within a single system, giving rise to rich quantum correlations [106, 107, 112, 117, 118, 119]. In *dI/dV* spectra, such interplay can manifest as the simultaneous appearance of Kondo resonances, inelastic spin‐excitation steps, and in‐gap YSR peaks. A comprehensive understanding of the coexistence and interplay among these effects is essential for elucidating the microscopic mechanisms of spin screening, magnetic excitation, and spin–superconductivity coupling at the molecular scale.

**FIGURE 5:**
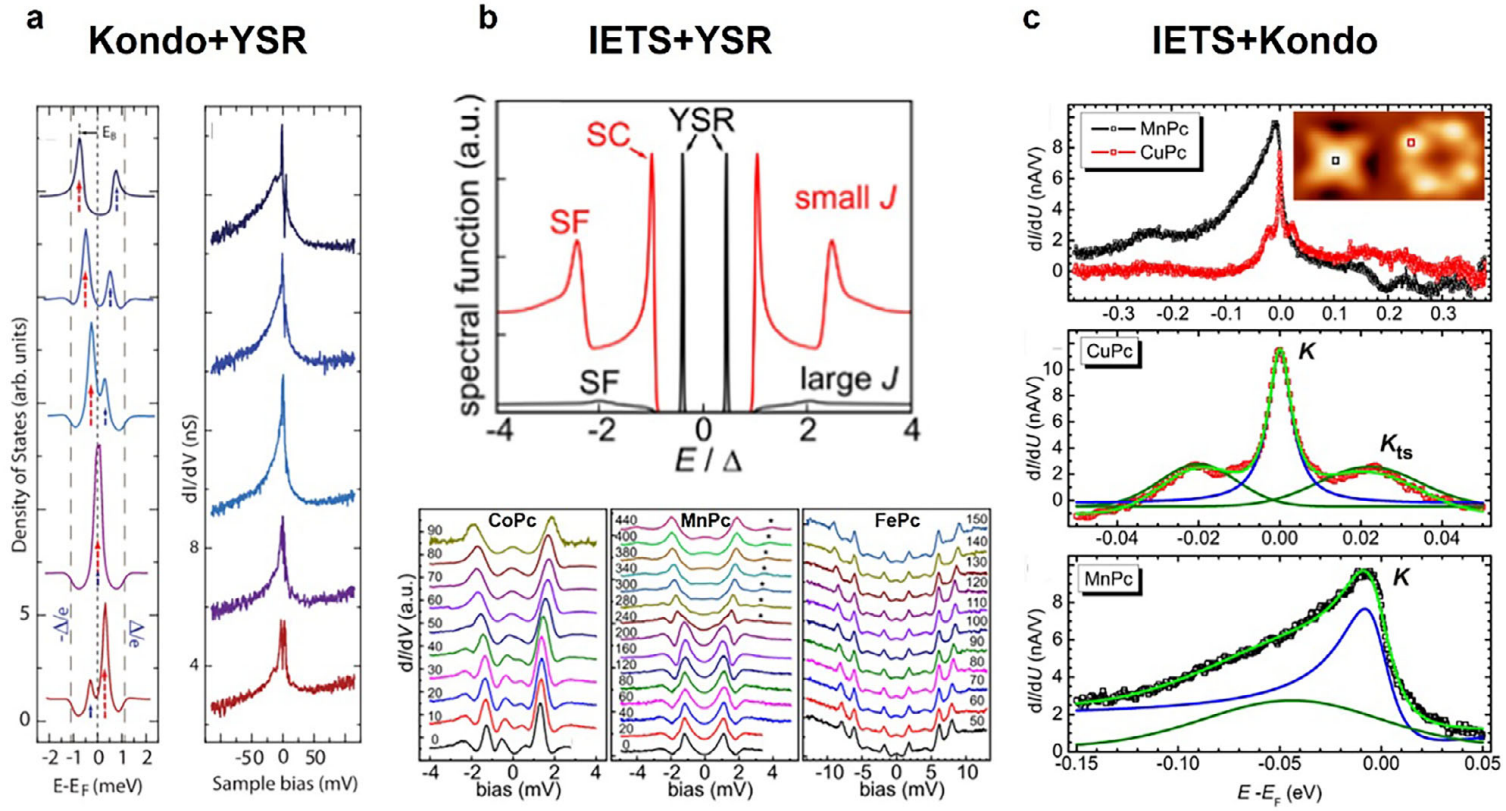
Coexistence of Kondo, IETS, and YSR states. (a) Left panel: low‐energy *dI/dV* spectra acquired on five characteristic MnPc molecules. Right panel: high‐energy *dI/dV* spectra acquired on the same molecules. Adapted with permission [112]. Copyright 2011, The American Association for the Advancement of Science. (b) Upper panel: impurity‐induced pairs of bound states symmetric with respect to *E_F_* within the quasiparticle excitation gap (black curve). Even at vanishing *J*, for impurities with *S* ≥ 1, the internal spin degrees of freedom can result in symmetric features with respect to the Fermi level outside the superconducting gap (red curve). Bottom panel: normalized *dI/dV* spectra recorded over CoPc, MnPc, FePc molecules at different tip−sample distances. As the tip is brought closer to the molecule (from bottom to top), the exchange coupling between the molecule and substrate becomes weaker. The initial tip−sample distance is set by the set‐point conditions, after which the tip is approached by a distance of *z*_offset_, as indicated in the panel. Adapted under the terms of the Creative Commons CC BY license [106]. Copyright 2019, American Chemical Society. (c) *dI/dV* spectra of MnPc and CuPc measured on the Ag(001) substrate at the marked positions. Inset: corresponding Kondo maps of the zero‐bias *dI/dV* spatial distributions. Adapted with permission [118]. Copyright 2014, American Chemical Society.

A prototypical example is the MnPc/Pb(111) system. An adsorbed MnPc molecule carries a spin *S* = 1 and couples to substrate electrons via two Kondo screening channels with distinct energy scales. One is a high‐energy broad‐band channel with a Kondo temperature *T_K _* ∼ 200–400 K [120, 121]. Because its characteristic energy scale greatly exceeds the superconducting gap Δ, the associated spin screening is essentially completed prior to the superconducting transition. The second is a low‐energy narrow‐band channel with a characteristic energy scale *k_B_T_K_*  ∼  Δ, where *k_B_* is the Boltzmann constant, which competes directly with the superconducting pairing below the superconducting critical temperature *T_C_*, thereby forming YSR bound states within the superconducting gap (Figure 5a left). More quantitatively, the coupling regimes can be classified by comparing the Kondo energy scale *k_B_T_K_* with the superconducting gap Δ. The Kondo‐dominant regime corresponds to *k_B_T_K_*  ≫  Δ, while the YSR‐dominant regime is realized for *k_B_T*_*K*  _ ≪  Δ. A crossover occurs at *k_B_T_K_*   ∼  Δ, where Kondo screening competes with Cooper pairing.

Above *T_C_*, at 8.8 K, all MnPc molecules exhibit zero‐bias conductance anomalies that can be fitted by two Fano line shapes with significantly different linewidths, revealing the existence of the two screening channels (Figure 5a). Measurements on the same molecules further show a strong correlation between the low‐energy scale *k_B_T_K_* at 8.8 K and the YSR bound‐state energy *E_B_* measured at 4.5 K: a weaker low‐energy Kondo channel corresponds to a YSR state deeper inside the gap, indicating stronger effective magnetic coupling and enhanced spin polarization [122]. When *k_B_T_K_*  ∼  Δ, the YSR state crosses the Fermi level, signaling a quantum phase transition. The ground state switches from a total singlet (*E_B _* <  0), consisting of a Kondo singlet coupled with weakly bound Cooper pairs, to a total doublet (*E_B_*   >  0), in which the low‐energy Kondo screening is incomplete and a broken Cooper‐pair spin (*S *= 1/2) is bound to an effective nonmagnetic impurity (*S *= 0). Overall, the high‐energy Kondo screening channel coexists with superconductivity on a high‐energy scale, while the low‐energy channel competes with the YSR bound states. The balance of this competition governs the quantum phase transition between a Kondo‐dominated singlet ground state and a YSR‐dominated doublet ground state [112].

On NbSe_2_, MnPc molecules (*S* = 3/2) exhibit a continuous crossover from YSR bound states to spin‐flip excitations as the exchange coupling with the superconducting substrate is reduced (Figure 5b). In the strong coupling regime, the spectra are dominated by symmetric in‐gap YSR resonances. Upon weakening the coupling, the YSR peaks progressively shift toward the superconducting coherence peaks and eventually evolve into symmetric spin‐flip excitations located outside the gap (Figure 5b top) [106]. Microscopically, the YSR bound state originates primarily from the longitudinal exchange term *JS_z_s_z_*, corresponding to spin‐dependent scattering of Bogoliubov quasiparticles from the magnetic impurity. Meanwhile, the spin‐flip excitations arise from the splitting of internal spin states induced by magnetic anisotropy, where the renormalization of the magnetic anisotropy is associated with the transverse part of the exchange interaction *J*(*S* ^+^ *s* ^−^ + *S* ^−^ *s* ^+^), in which *S* ^+^(*S* ^−^) and *s* ^+^(*s* ^−^) denote the raising (lowering) spin operators for the localized and conduction electron spins, respectively, describing mutual spin‐flip processes between them [123, 124]. Because NbSe_2_ exhibits a soft superconducting gap, lifetime broadening makes it difficult to resolve well‐defined YSR and spin‐flip features simultaneously for a single adsorption configuration. Nonetheless, this continuous spectral evolution is well reproduced by numerical renormalization‐group (NRG) calculations [125] based on an anisotropic Kondo model with *S* = 3/2, which reproduce the crossover between the YSR and spin‐excitation regimes. In comparison, FePc molecules (*S* = 1) on NbSe_2_ exhibit only spin‐flip excitations outside the superconducting gap and no discernible subgap YSR resonances, consistent with weaker exchange coupling and easy‐plane anisotropy. CoPc molecules (*S* = 1/2), meanwhile, display a pair of in‐gap YSR resonances that move toward the coherence peaks as the coupling strength is reduced (Figure 5b).

While MnPc is often regarded as a prototypical system due to its multi‐orbital nature and multiple Kondo screening channels, such coexistence phenomena are not exclusive to MnPc. When CuPc molecules are adsorbed on the Ag(001) surface, the *dI/dV* spectra show a pronounced zero‐bias resonance at the Fermi level, together with two finite‐bias side peaks (Figure 5c), indicating the coexistence of a Kondo resonance and IETS features [126]. This behavior is attributed to a ligand‐spin Kondo resonance, where the local magnetic moment is primarily hosted on the ligand π orbitals. The partially filled π‐derived states hybridize moderately with substrate conduction electrons, establishing an exchange coupling that produces the Kondo resonance. Meanwhile, tunneling electrons can drive intramolecular triplet–singlet magnetic excitations, giving rise to the inelastic side peaks [127]. By contrast, MnPc/Ag(001) exhibits a single broad Kondo resonance centered on the Mn ion, without discernible IETS features. This resonance mainly originates from the *d_z_^2^* orbital, whose strong coupling to the substrate underpins a high Kondo coherence temperature (∼120 K). Simultaneously, hybridization of the *d_yz/xz_* orbitals with ligand π states yields only a broad continuum near the Fermi level, lacking distinct inelastic or Kondo signatures. Together, these results demonstrate that in CuPc/Ag(001) the coexistence of Kondo and IETS reflects ligand‐mediated spin excitations, whereas in MnPc/Ag(001) Kondo screening is dominated by a single *d_z_^2^* orbital. Together, they represent distinct manifestations of multiorbital electronic correlations in TMPc molecules [118].

Overall, TMPc molecules adsorbed on different substrates can exhibit diverse spin‐related phenomena, including Kondo resonances, spin‐flip IETS features, and YSR states. These signatures originate from distinct microscopic mechanisms but may coexist or evolve continuously within a single system, depending on the local environment and relevant energy scales. Their spectral characteristics and underlying physical origins provide key information on spin–electron interactions at the molecule–substrate interface. A concise summary of their mechanisms and distinguishing features is provided in Table 1.

**TABLE 1:** Comparison of Kondo resonance, spin‐flip IETS, and YSR states.

	Kondo resonance	Spin‐flip IETS	YSR states	Coexistence/competition
Physical origin	Many‐body screening of a localized spin by conduction electrons	Inelastic tunneling–induced spin excitations	Magnetic‐impurity–induced bound states in a superconductor	Simultaneous coupling of a localized spin to conduction electrons and spin‐excitation/superconducting quasiparticle channels, with competing or coexisting scattering processes
dI/dV spectral signature	Peak or dip with characteristic Fano lineshape	Step‐like conductance changes are symmetric in bias	Particle–hole symmetric in‐gap resonances	Superposition or continuous evolution of spectral features
Energy range	Near the Fermi level (zero bias)	Finite bias corresponding to spin‐excitation energies	Inside the superconducting gap	Coexistence of zero‐bias anomalies, finite‐bias steps/peaks, and in‐gap bound states
Temperature dependence	Suppressed with increasing temperature; disappears for *T* > *T_K_*	Weak temperature dependence; thermal broadening of steps	Disappears above the superconducting critical temperature *T_c_*	Determined by the dominant energy scale (*T_K_* and *T_c_*)
Magnetic‐field response	Zeeman splitting of the Kondo resonance	Zeeman splitting of excitation thresholds	Field‐induced shift or splitting of YSR peaks	Distinct field responses of coexisting spectral features
Typical modeling approaches	Fano analysis; Anderson/Kondo impurity models	Effective spin Hamiltonian including magnetic anisotropy	Yu–Shiba–Rusinov impurity model	Multi‐orbital Anderson or anisotropic Kondo models
Representative TMPc systems	CoPc/Sb(111) [33]; FePc/Au(111) [32]; MnPc/Pb(111) [34]	FePc/Cu(110)(2×1)‐O [35]; CoPc/NbSe_2_ [36, 37, 161]	CoPc/NbSe_2_ [38]	MnPc/Pb(111) [112]; MnPc/NbSe_2_ [106]; CuPc/Ag(001) [118]

## 3 Manipulation of Magnetic Interactions in TMPc Adsorbates

### 3.1 Chemical Control of Spin States in TMPc Adsorbates

Chemical manipulation of spin states is a central strategy for tailoring magnetism at the atomic and molecular scales. The formation, rupture, or reconstruction of chemical bonds can directly modulate the electronic hybridization between a transition‐metal center and its local environment, thereby altering the energy ordering and occupation of the metal *d*‐orbitals and triggering transitions between different spin multiplicities [128, 129, 130, 131]. When processes such as dehydrogenation [58, 132], ligand exchange [57, 132, 133, 134], or surface adsorption [135, 136] modify the coupling strength between the metal center and its ligands or the underlying substrate, the extent of spin delocalization is correspondingly modified. These changes are typically reflected in magnetic‐moment reconstruction, the emergence or suppression of a Kondo resonance, and the redistribution of spin‐excitation energies. Thus, chemical control of spin states arises from a rebalancing between orbital‐hybridization strength and electronic filling, highlighting the intrinsic interplay between local electronic correlations and chemical bonding.

**FIGURE 6:**
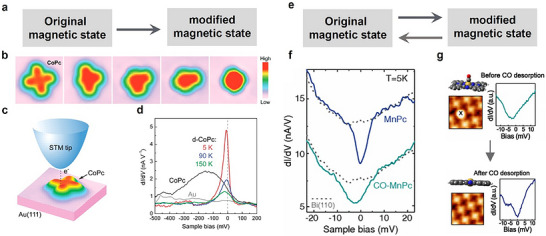
Reversible and irreversible chemical control of the spin states of TMPc adsorbates. (a,e) Schematic illustration of the irreversible and reversible chemical switching between the original and modified magnetic states of a TMPc molecule. (b) STM images showing the stepwise tip‐induced dehydrogenation process of a CoPc molecule on Au(111). (c) Diagram of the dehydrogenation induced by the tunneling current. (d) Typical *dI/dV* spectra measured at the centers of a CoPc molecule at 5 K (black line), showing a *dz^2^* OTM resonance, and a d‐CoPc molecule at 5, 90, and 150 K (colored lines), showing strong resonance near *E_F_*. Spectra from bare Au(111) (gray line) are shown for comparison. Adapted with permission [58]. Copyright 2005, The American Association for the Advancement of Science. (f) Low‐energy *dI/dV* spectra of MnPc and CO‐coordinated MnPc at 5 K, showing a zero bias anomaly. The dotted plots are the spectra measured on a bare Bi surface as a reference. (g) STM image and *dI/dV* spectra of the molecule before and after controlled CO desorption. Adapted with permission [135]. Copyright 2012, American Physical Society.

For intact CoPc molecules adsorbed on the Au(111) surface, the unpaired electron at the central Co atom is completely quenched due to molecule–substrate interactions. Accordingly, no Kondo resonance is observed in the *dI/dV* spectra (Figure 6d). By applying a local voltage pulse of approximately 3 V with an STM tip, the eight hydrogen atoms on the four indole rings can be sequentially removed, producing the dehydrogenated d‐CoPc species (Figure 6b,c). This dehydrogenation induces a pronounced structural rearrangement: peripheral carbon atoms move toward the Au substrate, while the central Co atom shifts upward, leading to a slight out‐of‐plane distortion of the molecular framework. The modified geometry strengthens molecule–surface electronic coupling, thereby reinstating a localized spin at the Co center and giving rise to a characteristic Kondo resonance in the *dI/dV* spectra (Figure 6d) [57]. Notably, although dehydrogenation recovers the local spin of CoPc and renders the Kondo resonance detectable in d‐CoPc, the process is irreversible, and the system cannot be returned to the original spin‐quenched state through the reverse reaction (Figure 6a).

In contrast, MnPc molecules enable reversible interconversion between distinct spin states via controlled coordination and desorption of CO (Figure 6e). On the Bi(110) surface, MnPc exhibits a narrow zero‐bias Kondo resonance (Figure 6f) arising from a partially screened local magnetic moment, corresponding to a magnetic ground state of *S* = 1. Upon coordination of a CO molecule to the central Mn ion, the zero‐bias resonance becomes markedly broadened (Figure 6g), indicating a further reduction of the effective spin to *S* = 1/2 and the emergence of a mixed‐valence regime dominated by charge fluctuations. This behavior can be attributed to CO acting as a strong‐field ligand, redistributing the Mn *d*‐orbital occupations and shifting their energies. Simultaneously, the relevant substrate coupling channels are modified (notably depleting the $d_{z^{2}}$ channel and leaving the *d_xy_* channel weakly hybridized near *E_F_*), thereby lowering the total spin and driving the system into the mixed‐valence state [137, 138, 139]. Crucially, this process is fully reversible: STM tip‐induced CO desorption restores both the pristine molecular contrast and the narrow Kondo resonance (Figure 6g), enabling reversible switching of MnPc on Bi(110) between the original *S* = 1 state and the ligand‐coordinated *S* = 1/2 state [135]. Chemical approaches tailor spin states by modifying bonding and coordination. In contrast, geometric or configurational changes enable control without altering the chemical composition.

### 3.2 Configuration‐Induced Spin States of TMPc Adsorbates

Variations in molecular configuration play a decisive role in modulating magnetic interactions at molecule‐substrate interfaces. The geometric structure of a molecule directly determines the spatial distribution and energy separation of its electronic orbitals [140, 141, 142, 143], thereby tuning key parameters such as crystal‐field splitting, orbital occupancy, and exchange interactions [144, 145]. Changes in configuration can alter the local coordination environment and overall symmetry, reorganizing spin‐related energy levels and inducing transitions between low‐spin and high‐spin states [146, 147, 148]. Such configuration‐driven spin regulation is an intrinsic structural effect that can operate without external stimuli, enabling precise control over spin behavior through subtle geometric adjustments. This intrinsic mechanism underscores the strong coupling between molecular geometry and electronic structure, providing a fundamental theoretical basis for the rational design of molecular systems with controllable spin characteristics.

**FIGURE 7:**
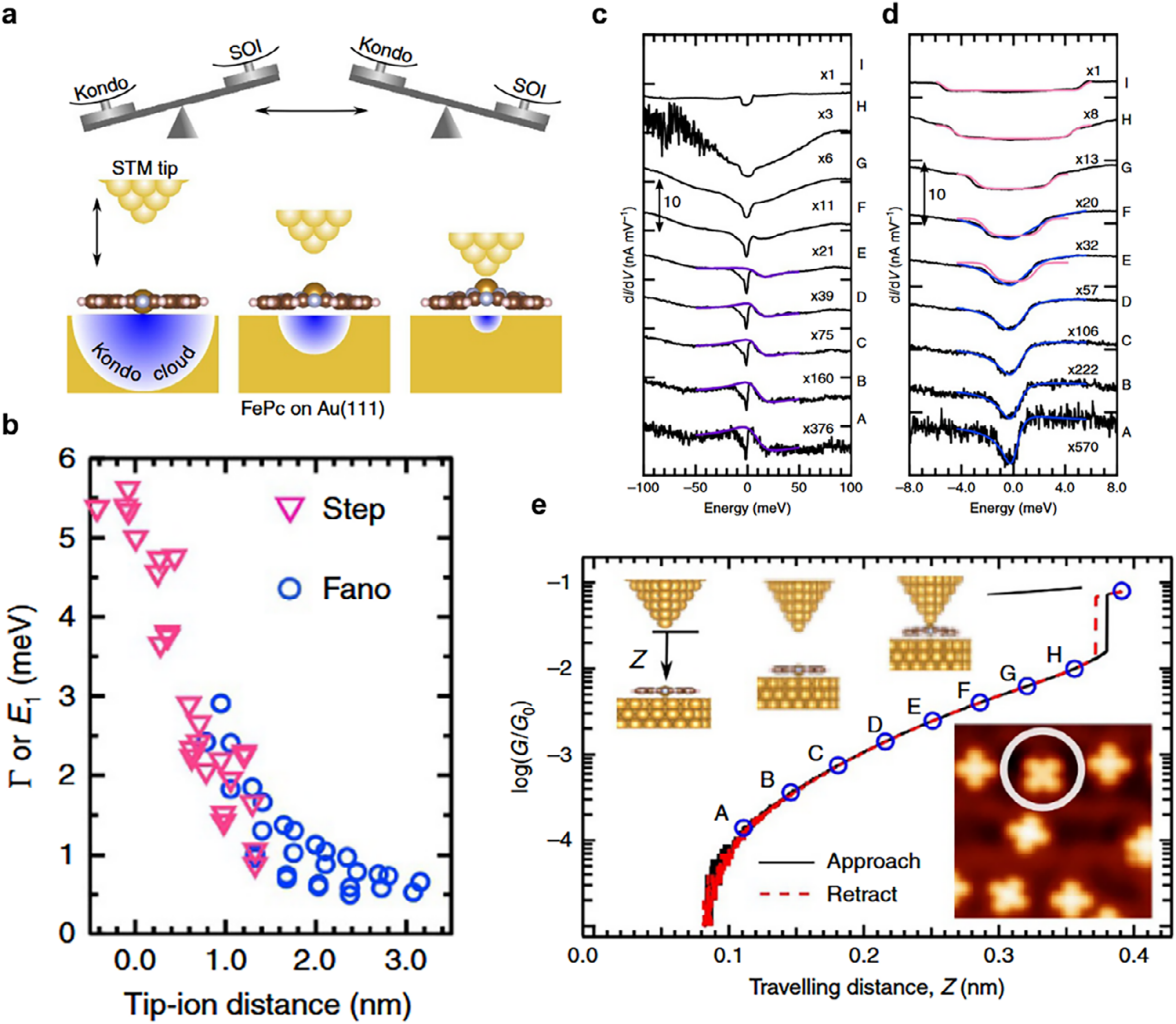
Configuration‐induced modulation of the spin state of FePc molecules on Au(111). (a) Schematic illustration of controlling the Kondo coupling by adjusting the STM tip position: lifting the Fe^2^^+^ ion in FePc/Au(111) transforms the molecule from a planar to a pyramidal configuration, weakens the hybridization with the Au substrate, and thereby tunes the Kondo exchange coupling to simulate the competition. (b) Transition of spectral feature from Fano‐Kondo type to a symmetric step structure. Variations of *Γ* and *E*_1_ are plotted with the tip‐ion distance, where *Γ* and *E*_1_ are the half‐width of the Fano‐Kondo resonance and the step energy, respectively. (c,d) Evolutions of tunnelling spectra in wide and narrow energy ranges measured at 0.4 K with approaching the STM tip to the Fe^2^^+^ ion. (e) Conductance trace measured as a function of the travelling distance of the tip. *G*_0_ is the conductance quantum. The conductance jump at *Z* = 0.39 nm indicates the contact of the tip and the molecule. Adapted under the terms of the Creative Commons CC BY license [149]. Copyright 2017, The Author(s).

In the FePc/Au(111) single‐molecule quantum‐dot system, the mechanical action of an STM tip offers precise control of molecular configuration and spin‐dependent electronic states [149]. As the tip is gradually brought closer to the Fe^2+^ ion within the ligand cage, the ion is pulled upward along the surface normal, distorting the molecule from a nearly planar geometry into a pyramidal configuration (Figure 7a). At the same time, the Fe^2+^‐Au distance increases and the hybridization between Fe *d*‐orbitals (particularly the *dz^2^*) and the metallic substrate is significantly reduced, leading to a continuous weakening of the Kondo coupling (Figure 7b). In the corresponding *dI/dV* spectra (Figure 7c–e), the broad peak and asymmetric Fano–Kondo dip near the Fermi level, originating from high and low Kondo temperature channels, gradually diminish and eventually vanish as the tip approaches. Meanwhile, the spectral lineshape evolves continuously from a characteristic Fano–Kondo resonance to a symmetric inelastic step‐like structure (Figure 7b). DFT calculations further show that, while the total spin *S* = 1 is preserved and the *d*‐orbital occupation remains essentially unchanged, the upward displacement of Fe^2+^ and the associated molecular reconstruction directly modify the hybridization between the *d*‐orbitals with the substrate and tip states. This lowers the Kondo temperature and weakens the Kondo screening, allowing the SOI‐induced level splitting to dominate the spectroscopic response [150, 151]. Therefore, the continuous evolution from a Fano–Kondo resonance to an SOI‐dominant inelastic step can be understood as a crossover from a Kondo‐dominant regime to an SOI‐dominant regime driven by tip‐induced configurational changes. Beyond intrinsic structural control, external stimuli offer additional ways to tune spin states. This enables reversible and real‐time manipulation under experimental conditions.

### 3.3 External‐Field Control of Spin States in TMPc Adsorbates

The spin states of TMPc molecules display exceptional sensitivity to external stimuli, making field control an essential and effective strategy for tailoring their magnetic properties on diverse substrates. Different external fields, including magnetic, electric, and thermal fields, can modulate the spin configuration, magnetic anisotropy, and spin coherence of TMPc molecules in distinct yet complementary manners.

**FIGURE 8:**
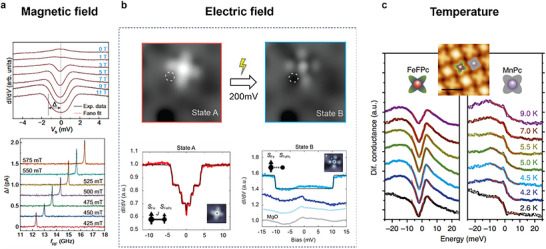
External‐field control of spin states in TMPc adsorbates. (a) Magnetic‐field control of spin states. Adapted with permission [152]. Copyright 2025, American Physical Society. (b) Electric‐field control of spin states. Adapted under the terms of the Creative Commons CC BY license [153]. Copyright 2025, The Author(s). (c) Temperature control of spin states. Adapted under the terms of the Creative Commons CC BY license [154]. Copyright 2017, The Author(s).

As shown in Figure 8a, applying an external magnetic field induces Zeeman splitting of the localized spin states in FePc, which gradually suppresses the Kondo resonance. This behavior originates from the lifting of spin degeneracy, which restricts spin‐flip scattering and thereby weakens the Kondo screening effect [152]. As illustrated in Figure 8b, electric‐field pulses delivered by the STM tip trigger a reversible transition between two bistable configurations of the Fe–FePc complex on MgO/Ag(001). The transition slightly modifies the registry of the Fe adatom relative to the FePc molecule, yielding distinct spin‐excitation spectra. These findings indicate that electric fields can modulate spin states via subtle rearrangements of the local atomic and electronic structure [153]. Figure 8c shows that increasing temperature progressively broadens and attenuates the Kondo resonance in FeFPc and MnPc, indicating that thermal fluctuations suppress spin coherence. As the temperature rises, Kondo screening becomes weakened, resulting in a reduction and broadening of the zero‐bias resonance. Temperature‐dependent *dI/dV* spectra further reveal a crossover from a coherent Kondo regime at low temperatures to a thermally broadened, incoherent regime at higher temperatures [154]. Collectively, these observations demonstrate that external fields play a crucial role in tuning the spin states of TMPc molecules, offering significant opportunities for molecular spintronic and quantum information devices.

Taken together, chemical modification, configurational control, and external‐field tuning provide complementary approaches for manipulating spin states in TMPc adsorbates. Chemical strategies enable direct and often robust modification of the electronic structure through bonding and coordination. Configurational control, in contrast, allows continuous tuning driven by geometric changes without altering the chemical composition. External fields further offer reversible and dynamic control under experimental conditions. A comparison of these approaches, including their mechanisms, reversibility, and operational regimes, is summarized in Table 2. Collectively, these complementary strategies establish a versatile framework for controlling spin states at the molecular scale, providing a solid foundation for the development of molecular spintronic and quantum devices.

**TABLE 2:** Comparison of spin‐state control mechanisms in TMPc adsorbates.

	Chemical control [58, 135]	Configurational control [149]	External‐field control [152, 153, 154]
Control mechanism	Modification of metal–ligand bonding and coordination	Structural distortion of the molecular framework	Application of magnetic, electric, or thermal fields
Electronic response	Variation in orbital hybridization and electron occupancy	Redistribution of orbital overlap and local crystal field	Zeeman splitting, field‐induced charge redistribution, thermal decoherence
Magnetic response	Transition between distinct spin states	Modulation of magnetic anisotropy and spin configuration	Continuous tuning of spin polarization and coherence
Reversibility	Predominantly irreversible; reversible in specific cases	Generally reversible	Intrinsically reversible
Spectroscopic manifestation	Emergence or suppression of Kondo resonance; shifts in spin‐excitation energies	Evolution of Kondo resonance and/or inelastic spin excitations	Zeeman splitting, suppression, and thermal broadening of Kondo features

## 4 Summary and Outlook

In recent years, STM has enabled atomic‐scale spatial resolution and spectroscopic capabilities for investigating magnetic interactions between TMPc molecules and substrates. This review summarizes key spin‐related phenomena observed in TMPc molecules on various substrates, including the Kondo effect [32, 33, 34], spin excitations [35, 36, 37], and YSR states [38, 103]. These findings highlight the complex interactions between localized molecular spins and the substrate electrons, and provide valuable insights into the coupling among spin, orbital, and charge degrees of freedom. Moreover, the coexistence and mutual correlation of Kondo resonances, IETS signals, and YSR states within single molecules highlight the richness of many‐body quantum effects and competing interactions in molecular‐scale spintronic systems [106, 112, 118].

The spin states in TMPc molecules on different substrates can be effectively tuned through chemical modification [58, 135], adsorption configuration [149], and external fields [152, 153, 154]. Ligand engineering and adsorption at the metal center enable control of the spin state and redistribution of charge density, while variations in adsorption geometry and molecule–substrate interaction can further modulate SOI and magnetic anisotropy. In addition, external stimuli such as magnetic field, electric field, and temperature can reversibly switch spin states, offering a platform for molecular‐scale spin switches and qubit implementations.

To date, research on the magnetic interactions between TMPc molecules and substrates has mainly focused on conventional three‐dimensional bulk metals. With the rapid emergence of 2D materials and strongly correlated electronic systems, attention is gradually shifting toward alternative substrates. Unlike conventional bulk metals, these substrates can exhibit reduced electronic screening, enhanced interfacial sensitivity, and emergent collective excitations, thereby offering new pathways for molecule–substrate spin interactions. In such environments, the magnetic behavior of TMPc molecules may arise not only from hybridization with itinerant electrons but also from correlation effects in the substrate, including Hubbard‐band formation, spin fluctuations, charge ordering, and symmetry‐broken electronic states. In addition, the exceptional tunability of two‐dimensional materials through layer number, stacking order, strain, and electrostatic gating provides a versatile platform for tuning exchange coupling, magnetic anisotropy, and Kondo screening at the single‐molecule level.

For instance, MnPc coupled to the correlated insulating phase of 1T‐NbSe_2_, which exhibits characteristics of a quantum spin liquid, has been reported to exhibit spinon Kondo resonance near the Hubbard band edges [155]. This result is particularly significant because it suggests that the screening of a localized molecular moment may involve many‐body excitations rather than only conventional conduction electrons. Consequently, the Kondo effect in such hybrid systems may exhibit unconventional spectral, spatial, and temperature‐dependent characteristics, providing an attractive platform for exploring how fractionalized or correlation‐driven excitations interact with localized molecular moments.

In addition, studies of TMPc molecules on 2D magnetic substrates, such as the prototypical ferromagnet CrI_3_, are rapidly advancing [156, 157]. These developments suggest that TMPc interactions with novel quantum materials may generate new phenomena driven by intricate many‐body effects. Compared with bulk magnetic metals, two‐dimensional magnets provide atomically thin magnetic layers and well‐defined interfaces, where molecule–substrate exchange interactions can be sensitive to stacking configuration, magnetic ordering, and the dielectric environment. This sensitivity may enable tunable modulation of interfacial spin alignment, spin‐flip scattering, and magnetic anisotropy, which is desirable for molecular‐scale spin filtering and readout. These systems may therefore offer a promising platform for exploring new phenomena driven by many‐body interactions.

Despite substantial progress, TMPc‐based systems still face important scientific and technological challenges. Leveraging ultra‐low‐temperature, ultra‐high‐vacuum STM platforms, future work could integrate advanced techniques such as SP‐STM [34, 152], electron spin resonance STM (ESR‐STM) [152, 153, 158, 159], and molecule‐functionalized‐tip STM (e.g., decorating the tip apex with a magnetic molecule) [160]. Importantly, these techniques offer complementary access to different aspects of interfacial magnetism. SP‐STM can probe the relative spin alignment between molecular and substrate moments and map the spatial distribution of spin polarization at the interface. ESR‐STM provides access to key spin Hamiltonian parameters, such as magnetic anisotropy and g‐factors, and enables studies of spin dynamics and coherent spin manipulation. Molecule‐functionalized tips may provide complementary sensitivity to spin‐dependent tunneling and local magnetic interactions, offering additional routes to investigate exchange coupling and spin dynamics. Together, these approaches may enable high‐resolution imaging and quantitative characterization of local spin orientation, exchange coupling, and spin dynamics, thereby providing key insights into the microscopic origins of molecular magnetism and its response to external perturbations.

Furthermore, while most existing studies emphasize how substrates affect TMPc spin states, the reverse influence of molecular spins on the substrate's electronic structure and magnetic order remains largely unexplored. Notably, recent work has shown that CoPc molecules can induce magnetism in NbSe_2_. This finding demonstrates that molecular spins can effectively modulate the electronic structure and magnetic response of the underlying substrate through interfacial coupling [161]. Building on this insight, future investigations should therefore address bidirectional spin coupling at molecule–substrate interfaces. Systems involving magnetic metals, superconductors, or topological materials are particularly promising for elucidating how molecular spins modify local magnetization, exchange interactions, and spin transport. Such efforts will deepen our understanding of spin communication across interfaces and support the development of molecular spintronic devices with tunable interfacial magnetism.

## Conflicts of Interest

The authors declare no conflicts of interest.

## Data Availability

The authors have nothing to report.

## References

[1] P. Wang , F. Chen , Y. Yang , et al., “Orbitronics: Mechanisms, Materials and Devices,” Advanced Electronic Materials 11 (2025): 2400554, 10.1002/aelm.202400554.

[2] Y. Guo , X. Zhang , Z. Huang , et al., “Quantum Materials for Spintronic Applications,” npj Spintronics 2 (2024): 36, 10.1038/s44306-024-00038-z.

[3] V. D. Nguyen , S. Rao , K. Wostyn , and S. Couet , “Recent Progress in Spin‐Orbit Torque Magnetic Random‐Access Memory,” npj Spintronics 2 (2024): 48, 10.1038/s44306-024-00044-1.

[4] S. Shi , X. Wang , Y. Zhao , and W. Zhao , “Recent Progress in Strong Spin‐Orbit Coupling van der Waals Materials and Their Heterostructures for Spintronic Applications,” Materials Today Electronics 6 (2023): 100060, 10.1016/j.mtelec.2023.100060.

[5] D. Jo , D. Go , G.‐M. Choi , and H.‐W. Lee , “Spintronics Meets Orbitronics: Emergence of Orbital Angular Momentum in Solids,” npj Spintronics 2 (2024): 19, 10.1038/s44306-024-00023-6.

[6] S. Loth , S. Baumann , C. P. Lutz , D. M. Eigler , and A. J. Heinrich , “Bistability in Atomic‐Scale Antiferromagnets,” Science 335 (2012): 196–199, 10.1126/science.1214131. 22246771

[7] M. Z. Hasan and C. L. Kane , “Colloquium: Topological Insulators,” Reviews of Modern Physics 82 (2010): 3045–3067, 10.1103/RevModPhys.82.3045.

[8] J. T. Mäkinen , S. Autti , and V. B. Eltsov , “Magnon Bose–Einstein Condensates: From Time Crystals and Quantum Chromodynamics to Vortex Sensing and Cosmology,” Applied Physics Letters 124 (2024): 100502, 10.1063/5.0189649.

[9] D. Loss and D. P. DiVincenzo , “Quantum Computation With Quantum Dots,” Physical Review A 57 (1998): 120–126, 10.1103/PhysRevA.57.120.

[10] D. D. Awschalom , L. C. Bassett , A. S. Dzurak , E. L. Hu , and J. R. Petta , “Quantum Spintronics: Engineering and Manipulating Atom‐Like Spins in Semiconductors,” Science 339 (2013): 1174–1179, 10.1126/science.1231364. 23471400

[11] C. Chappert , A. Fert , and F. N. Van Dau , “The Emergence of Spin Electronics in Data Storage,” Nature Materials 6 (2007): 813–823, 10.1038/nmat2024. 17972936

[12] R. Hanson , L. P. Kouwenhoven , J. R. Petta , S. Tarucha , and L. M. K. Vandersypen , “Spins in Few‐Electron Quantum Dots,” Reviews of Modern Physics 79 (2007): 1217–1265, 10.1103/RevModPhys.79.1217.

[13] B. H. Rimmler , B. K. Hazra , B. Pal , et al., “Atomic Displacements Enabling the Observation of the Anomalous Hall Effect in a Non‐Collinear Antiferromagnet,” Advanced Materials 35 (2023): 2209616, 10.1002/adma.202209616. 36996804

[14] Y. Wu , B. Peng , Z. Zeng , et al., “Voltage‐Controlled Topological Spin Textures in the Monolayer Limit,” Nature Communications 17 (2026): 2923, 10.1038/s41467-026-69800-7. PMC13031274 41708663

[15] Z. Shen , L. She , Y. Yang , et al., “Tuning the Quantum Spin States of Co‐Phthalocyanine on Good, Semi‐, and Half‐Metals,” Physical Review B 110 (2024): 174407, 10.1103/PhysRevB.110.174407.

[16] T. Pei , J. O. Thomas , S. Sopp , et al., “Exchange‐Induced Spin Polarization in a Single Magnetic Molecule Junction,” Nature Communications 13 (2022): 4506, 10.1038/s41467-022-31909-w. PMC9349289 35922414

[17] C. Yang , H. Wu , J. Cao , and X. Guo , “Mechanisms and Control of Spin Interactions in Molecular‐Scale Spintronics,” Newton 1 (2025): 100170, 10.1016/j.newton.2025.100170.

[18] F. Frezza , M. Kumar , A. Sánchez‐Grande , et al., “On‐Surface Synthesis of a Large‐Scale 2D MOF With Competing π–d Ferromagnetic/Antiferromagnetic Order,” Journal of the American Chemical Society 147 (2025): 19575–19582, 10.1021/jacs.4c17993. 40445041 PMC12164333

[19] Q. Du , X. Su , Y. Liu , et al., “Orbital‐Symmetry Effects on Magnetic Exchange in Open‐Shell Nanographenes,” Nature Communications 14 (2023): 4802, 10.1038/s41467-023-40542-0. PMC10412602 37558678

[20] M. Serri , W. Wu , L. R. Fleet , et al., “High‐Temperature Antiferromagnetism in Molecular Semiconductor Thin Films and Nanostructures,” Nature Communications 5 (2014): 3079, 10.1038/ncomms4079. PMC3941018 24445992

[21] G. Chiappe and E. Louis , “Kondo Effect of an Adsorbed Cobalt Phthalocyanine (CoPc) Molecule: The Role of Quantum Interference,” Physical Review Letters 97 (2006): 076806, 10.1103/PhysRevLett.97.076806. 17026264

[22] R. Wilhelmer , M. Diez , J. K. Krondorfer , and A. W. Hauser , “Molecular Pseudorotation in Phthalocyanines as a Tool for Magnetic Field Control at the Nanoscale,” Journal of the American Chemical Society 146 (2024): 14620–14632, 10.1021/jacs.4c01915. 38743819 PMC11140746

[23] M. Warner , S. Din , I. S. Tupitsyn , et al., “Potential for Spin‐Based Information Processing in a Thin‐Film Molecular Semiconductor,” Nature 503 (2013): 504–508, 10.1038/nature12597. 24162849

[24] A. Atxabal , M. Ribeiro , S. Parui , et al., “Spin Doping Using Transition Metal Phthalocyanine Molecules,” Nature Communications 7 (2016): 13751, 10.1038/ncomms13751. PMC5159905 27941810

[25] Y.‐J. Yang , S.‐X. Li , D.‐L. Chen , and Z.‐W. Long , “Geometric Structure, Electronic, and Spectral Properties of Metal‐Free Phthalocyanine Under the External Electric Fields,” ACS Omega 7 (2022): 41266–41274, 10.1021/acsomega.2c04941. 36406576 PMC9670904

[26] E. Annese , T. J. A. Mori , P. Schio , B. R. Salles , and J. C. Cezar , “Hybrid Organic/Inorganic Interface: In Situ and Ex Situ Characterization in Terms of Structure, Morphology, Electronic and Magnetic Properties,” Journal of Magnetism and Magnetic Materials 584 (2023): 171050, 10.1016/j.jmmm.2023.171050.

[27] R. R. Cranston and B. H. Lessard , “Metal Phthalocyanines: Thin‐Film Formation, Microstructure, and Physical Properties,” RSC Advances 11 (2021): 21716–21737, 10.1039/D1RA03853B. 35478816 PMC9034105

[28] E. B. Boydas and M. Roemelt , “The Trials and Triumphs of Modelling X‐Ray Absorption Spectra of Transition Metal Phthalocyanines,” Physical Chemistry Chemical Physics 26 (2024): 20376–20387, 10.1039/D4CP01900H. 39015952

[29] M.‐S. Liao and S. Scheiner , “Electronic Structure and Bonding in Metal Phthalocyanines, Metal=Fe, Co, Ni, Cu, Zn, Mg, Metal = Fe, Co, Ni, Cu, Zn, Mg,” Journal of Chemical Physics 114 (2001): 9780–9791, 10.1063/1.1367374.

[30] N. Tsukahara , M. Kawai , and N. Takagi , “Impact of Reduced Symmetry on Magnetic Anisotropy of a Single Iron Phthalocyanine Molecule on a Cu Substrate,” Journal of Chemical Physics 144 (2016): 044701, 10.1063/1.4940138. 26827222

[31] J. Fernández‐Rodríguez , B. Toby , and M. Van Veenendaal , “Mixed Configuration Ground state in Iron(II) Phthalocyanine,” Physical Review B 91 (2015): 214427, 10.1103/PhysRevB.91.214427.

[32] N. Tsukahara , S. Shiraki , S. Itou , N. Ohta , N. Takagi , and M. Kawai , “Evolution of Kondo Resonance From a Single Impurity Molecule to the Two‐Dimensional Lattice,” Physical Review Letters 106 (2011): 187201, 10.1103/PhysRevLett.106.187201. 21635122

[33] L. She , Z. Shen , Z. Xie , et al., “Magnetic Moment Preservation and Emergent Kondo Resonance of Co‐Phthalocyanine on Semimetallic Sb(111),” Physical Review Letters 129 (2022): 026802, 10.1103/PhysRevLett.129.026802. 35867437

[34] Y.‐S. Fu , Q.‐K. Xue , and R. Wiesendanger , “Spin‐Resolved Splitting of Kondo Resonances in the Presence of RKKY‐Type Coupling,” Physical Review Letters 108 (2012): 087203, 10.1103/PhysRevLett.108.087203. 22463564

[35] N. Tsukahara , K.‐I. Noto , M. Ohara , et al., “Adsorption‐Induced Switching of Magnetic Anisotropy in a Single Iron(II) Phthalocyanine Molecule on an Oxidized Cu(110) Surface,” Physical Review Letters 102 (2009): 167203, 10.1103/PhysRevLett.102.167203. 19518750

[36] Y. Wang , S. Arabi , K. Kern , and M. Ternes , “Symmetry Mediated Tunable Molecular Magnetism on a 2D Material,” Communications Physics 4 (2021): 103, 10.1038/s42005-021-00601-8.

[37] R. Drost , S. Kezilebieke , J. L. Lado , and P. Liljeroth , “Real‐Space Imaging of Triplon Excitations in Engineered Quantum Magnets,” Physical Review Letters 131 (2023): 086701, 10.1103/PhysRevLett.131.086701. 37683177

[38] S. Kezilebieke , M. Dvorak , T. Ojanen , and P. Liljeroth , “Coupled Yu–Shiba–Rusinov States in Molecular Dimers on NbSe_2_ ,” Nano Letters 18 (2018): 2311–2315, 10.1021/acs.nanolett.7b05050. 29533636 PMC6095633

[39] X. Li , L. Zhu , A. Zhao , et al., “Co and CoPc Molecular Kondo Box on Gold Surface,” Physical Review Letters 135 (2025): 086201, 10.1103/65qq-dknn. 40929313

[40] D.‐J. Choi , R. Robles , S. Yan , et al., “Building Complex Kondo Impurities by Manipulating Entangled Spin Chains,” Nano Letters 17 (2017): 6203–6209, 10.1021/acs.nanolett.7b02882. 28872317

[41] Y. Li , A. T. Ngo , A. DiLullo , et al., “Anomalous Kondo Resonance Mediated by Semiconducting Graphene Nanoribbons in a Molecular Heterostructure,” Nature Communications 8 (2017): 946, 10.1038/s41467-017-00881-1. PMC5643342 29038513

[42] F. Ara , S. M. Fakruddin Shahed , M. I. Hossain , K. Katoh , M. Yamashita , and T. Komeda , “Control of the Magnetic Interaction Between Single‐Molecule Magnet TbPc_2_ and Superconductor NbSe_2_ Surface by an Intercalated Co Atom,” Nano Letters 23 (2023): 6900–6906, 10.1021/acs.nanolett.3c01298. 37505070

[43] X. Meng , J. Möller , M. Mansouri , et al., “Controlling the Spin States of FeTBrPP on Au(111),” ACS Nano 17 (2023): 1268–1274, 10.1021/acsnano.2c09310. 36440841 PMC10711789

[44] L. Farinacci , G. Ahmadi , M. Ruby , et al., “Interfering Tunneling Paths Through Magnetic Molecules on Superconductors: Asymmetries of Kondo and Yu‐Shiba‐Rusinov Resonances,” Physical Review Letters 125 (2020): 256805, 10.1103/PhysRevLett.125.256805. 33416394

[45] X. Meng , J. Möller , R. E. Menchón , et al., “Kondo Effect of Co‐Porphyrin: Remarkable Sensitivity to Adsorption Sites and Orientations,” Nano Letters 24 (2024): 180–186, 10.1021/acs.nanolett.3c03669. 38150551

[46] Y. Gao , S. Vlaic , T. Gorni , et al., “Manipulation of the Magnetic State of a Porphyrin‐Based Molecule on Gold: From Kondo to Quantum Nanomagnet via the Charge Fluctuation Regime,” ACS Nano 17 (2023): 9082–9089, 10.1021/acsnano.2c12223. 37162317

[47] D. M. Eigler and E. K. Schweizer , “Positioning Single Atoms With a Scanning Tunnelling Microscope,” Nature 344 (1990): 524–526, 10.1038/344524a0.

[48] Ø. Fischer , M. Kugler , I. Maggio‐Aprile , C. Berthod , and C. Renner , “Scanning Tunneling Spectroscopy of High‐Temperature Superconductors,” Reviews of Modern Physics 79 (2007): 353–419, 10.1103/RevModPhys.79.353.

[49] G. Binnig , H. Rohrer , C. Gerber , and E. Weibel , “Surface Studies by Scanning Tunneling Microscopy,” Physical Review Letters 49 (1982): 57–61, 10.1103/PhysRevLett.49.57.

[50] A. A. Aligia , “Low‐Energy Physics for an Iron Phthalocyanine Molecule on Au(111),” Physical Review B 105 (2022): 205114, 10.1103/PhysRevB.105.205114.

[51] C. Tresca , T. Bilgeri , G. Ménard , et al., “Importance of Accurately Measuring LDOS Maps Using Scanning Tunneling Spectroscopy in Materials Presenting Atom‐Dependent Charge Order: The Case of the Correlated Pb/Si(111) Single Atomic Layer,” Physical Review B 107 (2023): 035125, 10.1103/PhysRevB.107.035125.

[52] A. Odobesko , R. L. Klees , F. Friedrich , E. M. Hankiewicz , and M. Bode , “Boosting Spatial and Energy Resolution in STM With a Double‐Functionalized Probe,” Science Advances 10 (2024): adq6975, 10.1126/sciadv.adq6975. PMC11352829 39196925

[53] J. Kondo , “Resistance Minimum in Dilute Magnetic Alloys,” Progress of Theoretical Physics 32 (1964): 37–49, 10.1143/PTP.32.37.

[54] R. C. de Campos Ferreira , A. Sagwal , J. Doležal , et al., “Resonant Tip‐Enhanced Raman Spectroscopy of a Single‐Molecule Kondo System,” ACS Nano 18 (2024): 13164–13170, 10.1021/acsnano.4c02105. 38711331 PMC11112976

[55] V. Madhavan , W. Chen , T. Jamneala , M. F. Crommie , and N. S. Wingreen , “Tunneling into a Single Magnetic Atom: Spectroscopic Evidence of the Kondo Resonance,” Science 280 (1998): 567–569, 10.1126/science.280.5363.567. 9554843

[56] A. A. Khajetoorians , M. Steinbrecher , M. Ternes , et al., “Tailoring the Chiral Magnetic Interaction Between Two Individual Atoms,” Nature Communications 7 (2016): 10620, 10.1038/ncomms10620. PMC4766390 26902332

[57] E. Minamitani , N. Tsukahara , D. Matsunaka , Y. Kim , N. Takagi , and M. Kawai , “Symmetry‐Driven Novel Kondo Effect in a Molecule,” Physical Review Letters 109 (2012): 086602, 10.1103/PhysRevLett.109.086602. 23002765

[58] A. Zhao , Q. Li , L. Chen , et al., “Controlling the Kondo Effect of an Adsorbed Magnetic Ion Through Its Chemical Bonding,” Science 309 (2005): 1542–1544, 10.1126/science.1113449. 16141069

[59] S. Bhandary , E. Poli , G. Teobaldi , and D. D. O'Regan , “Dynamical Screening of Local Spin Moments at Metal–Molecule Interfaces,” ACS Nano 17 (2023): 5974–5983, 10.1021/acsnano.3c00247. 36881865 PMC10062023

[60] A. Mugarza , R. Robles , C. Krull , R. Korytár , N. Lorente , and P. Gambardella , “Electronic and Magnetic Properties of Molecule‐Metal Interfaces: Transition‐Metal Phthalocyanines Adsorbed on Ag(100),” Physical Review B 85 (2012): 155437, 10.1103/PhysRevB.85.155437.

[61] J. Uihlein , M. Polek , M. Glaser , et al., “Influence of Graphene on Charge Transfer Between CoPc and Metals: The Role of Graphene–Substrate Coupling,” Journal of Physical Chemistry C 119 (2015): 15240–15247, 10.1021/acs.jpcc.5b02912.

[62] D. Jacob , M. Soriano , and J. J. Palacios , “Kondo Effect and Spin Quenching in High‐Spin Molecules on Metal Substrates,” Physical Review B 88 (2013): 134417, 10.1103/PhysRevB.88.134417.

[63] P. Wahl , P. Simon , L. Diekhöner , et al., “Exchange Interaction Between Single Magnetic Adatoms,” Physical Review Letters 98 (2007): 056601, 10.1103/PhysRevLett.98.056601. 17358878

[64] P. Simon , R. López , and Y. Oreg , “Ruderman‐Kittel‐Kasuya‐Yosida and Magnetic‐Field Interactions in Coupled Kondo Quantum Dots,” Physical Review Letters 94 (2005): 086602, 10.1103/PhysRevLett.94.086602. 15783913

[65] W. Wan , R. Harsh , A. Meninno , et al., “Evidence for Ground State Coherence in a Two‐Dimensional Kondo Lattice,” Nature Communications 14 (2023): 7005, 10.1038/s41467-023-42803-4. PMC10622499 37919299

[66] K. P. Wójcik and J. Kroha , “Asymmetry Effects on the Phases of RKKY‐Coupled Two‐Impurity Kondo Systems,” Physical Review B 107 (2023): 125146, 10.1103/PhysRevB.107.125146.

[67] Y. Yayon , V. W. Brar , L. Senapati , S. C. Erwin , and M. F. Crommie , “Observing Spin Polarization of Individual Magnetic Adatoms,” Physical Review Letters 99 (2007): 067202, 10.1103/PhysRevLett.99.067202. 17930864

[68] R. Wiesendanger , “Spin Mapping at the Nanoscale and Atomic Scale,” Reviews of Modern Physics 81 (2009): 1495–1550, 10.1103/RevModPhys.81.1495.

[69] C. Iacovita , M. V. Rastei , B. W. Heinrich , et al., “Visualizing the Spin of Individual Cobalt‐Phthalocyanine Molecules,” Physical Review Letters 101 (2008): 116602, 10.1103/PhysRevLett.101.116602. 18851307

[70] Q. Zhuang , X. Wang , L. Ye , Y. Yan , and X. Zheng , “Origin of Asymmetric Splitting of Kondo Peak in Spin‐Polarized Scanning Tunneling Spectroscopy: Insights From First‐Principles‐Based Simulations,” Journal of Physical Chemistry Letters 13 (2022): 2094–2100, 10.1021/acs.jpclett.2c00228. 35225612

[71] M. Bagchi , T. Y. Tounsi , A. Safeer , et al., “Spin‐Polarized Scanning Tunneling Microscopy Measurements of an Anderson Impurity,” Physical Review Letters 133 (2024): 246701, 10.1103/PhysRevLett.133.246701. 39750358

[72] B. Fischer and M. W. Klein , “Magnetic and Nonmagnetic Impurities in Two‐Dimensional Metals,” Physical Review B 11 (1975): 2025–2029, 10.1103/PhysRevB.11.2025.

[73] P. Ferriani , C. Lazo , and S. Heinze , “Origin of the Spin Polarization of Magnetic Scanning Tunneling Microscopy Tips,” Physical Review B 82 (2010): 054411, 10.1103/PhysRevB.82.054411.

[74] J. Hieulle , S. Castro , N. Friedrich , et al., “On‐Surface Synthesis and Collective Spin Excitations of a Triangulene‐Based Nanostar,” Angewandte Chemie International Edition 60 (2021): 25224–25229, 10.1002/anie.202108301. 34647398 PMC9292598

[75] C. F. Hirjibehedin , C.‐Y. Lin , A. F. Otte , et al., “Large Magnetic Anisotropy of a Single Atomic Spin Embedded in a Surface Molecular Network,” Science 317 (2007): 1199–1203, 10.1126/science.1146110. 17761877

[76] C. F. Hirjibehedin , C. P. Lutz , and A. J. Heinrich , “Spin Coupling in Engineered Atomic Structures,” Science 312 (2006): 1021–1024, 10.1126/science.1125398. 16574821

[77] J. Fernández‐Rossier , “Theory of Single‐Spin Inelastic Tunneling Spectroscopy,” Physical Review Letters 102 (2009): 256802, 10.1103/PhysRevLett.102.256802. 19659108

[78] J. C. G. Henriques , C. Zhao , G. Catarina , P. Ruffieux , R. Fasel , and J. Fernández‐Rossier , “Determining Energy Dispersion of Spin Excitations With Scanning Tunneling Spectroscopy,” Physical Review Letters 135 (2025): 096703, 10.1103/n8jn-p468. 40952205

[79] H. Song , “Inelastic Electron Tunneling Spectroscopy of Molecular Electronic Junctions: Recent Advances and Applications,” Crystals 15 (2025): 681, 10.3390/cryst15080681.

[80] F. Delgado and J. Fernández‐Rossier , “Cotunneling Theory of Atomic Spin Inelastic Electron Tunneling Spectroscopy,” Physical Review B 84 (2011): 045439, 10.1103/PhysRevB.84.045439. 21902416

[81] Y.‐S. Fu , T. Zhang , S.‐H. Ji , et al., “Identifying Charge States of Molecules With Spin‐Flip Spectroscopy,” Physical Review Letters 103 (2009): 257202, 10.1103/PhysRevLett.103.257202. 20366279

[82] E. Minamitani , N. Takagi , and S. Watanabe , “Model Hamiltonian Approach to the Magnetic Anisotropy of Iron Phthalocyanine at Solid Surfaces,” Physical Review B 94 (2016): 205402, 10.1103/PhysRevB.94.205402.

[83] X. Chen , Y.‐S. Fu , S.‐H. Ji , et al., “Probing Superexchange Interaction in Molecular Magnets by Spin‐Flip Spectroscopy and Microscopy,” Physical Review Letters 101 (2008): 197208, 10.1103/PhysRevLett.101.197208. 19113306

[84] Y.‐S. Fu , S.‐H. Ji , T. Zhang , et al., “Ultrathin Lead Oxide Film on Pb(111) and Its Application in Single Spin Detection,” Applied Physics Letters 95 (2009): 063107, 10.1063/1.3205118.

[85] A. Abragam and B. Bleaney , Electron Paramagnetic Resonance of Transition Ions (Oxford University Press, 2012).

[86] T. Biktagirov , W. G. Schmidt , and U. Gerstmann , “Calculation of Spin‐Spin Zero‐Field Splitting Within Periodic Boundary Conditions: Towards All‐Electron Accuracy,” Physical Review B 97 (2018): 115135, 10.1103/PhysRevB.97.115135.

[87] J. H. Van Vleck , “On the Anisotropy of Cubic Ferromagnetic Crystals,” Physical Review 52 (1937): 1178–1198, 10.1103/PhysRev.52.1178.

[88] R. Boča , “Zero‐Field Splitting in Metal Complexes,” Coordination Chemistry Reviews 248 (2004): 757–815, 10.1016/j.ccr.2004.03.001.

[89] D. Wang , X. Bo , F. Tang , and X. Wan , “First‐Principles Study of the Spin‐Orbit Coupling Contribution to Anisotropic Magnetic Interactions,” Physical Review B 108 (2023): 085140, 10.1103/PhysRevB.108.085140.

[90] A. Köhn and J. Netz , “Treatment of Spin–Orbit Coupling With Internally Contracted Multireference Coupled Cluster Theory,” Physical Chemistry Chemical Physics 27 (2025): 13861–13869, 10.1039/D5CP01698C. 40530701

[91] M. Ternes , “Probing Magnetic Excitations and Correlations in Single and Coupled Spin Systems With Scanning Tunneling Spectroscopy,” Progress in Surface Science 92 (2017): 83–115, 10.1016/j.progsurf.2017.01.001.

[92] S. Notbohm , P. Ribeiro , B. Lake , et al., “One‐ and Two‐Triplon Spectra of a Cuprate Ladder,” Physical Review Letters 98 (2007): 027403, 10.1103/PhysRevLett.98.027403. 17358648

[93] P. A. McClarty , F. Krüger , T. Guidi , et al., “Topological Triplon Modes and Bound States in a Shastry–Sutherland Magnet,” Nature Physics 13 (2017): 736–741, 10.1038/nphys4117.

[94] Y. Luh , “Bound State in Superconductors With Paramagnetic Impurities,” Acta Physica Sinica 21 (1965): 75–91, 10.7498/aps.21.75.

[95] H. Shiba , “Classical Spins in Superconductors,” Progress of Theoretical Physics 40 (1968): 435–451, 10.1143/PTP.40.435.

[96] A. I. Rusinov , “Superconductivity Near a Paramagnetic Impurity,” Soviet Journal of Experimental and Theoretical Physics Letters 9 (1969): 85.

[97] D. Wang , J. Wiebe , R. Zhong , G. Gu , and R. Wiesendanger , “Spin‐Polarized Yu‐Shiba‐Rusinov States in an Iron‐Based Superconductor,” Physical Review Letters 126 (2021): 076802, 10.1103/PhysRevLett.126.076802. 33666492

[98] S. Karan , H. Huang , C. Padurariu , et al., “Superconducting Quantum Interference at the Atomic Scale,” Nature Physics 18 (2022): 893–898, 10.1038/s41567-022-01644-6.

[99] S. Karan , H. Huang , A. Ivanovic , et al., “Tracking a Spin‐Polarized Superconducting Bound State Across a Quantum Phase Transition,” Nature Communications 15 (2024): 459, 10.1038/s41467-024-44708-2. PMC10784290 38212303

[100] H. Huang , S. Karan , C. Padurariu , et al., “Universal Scaling of Tunable Yu‐Shiba‐Rusinov States Across the Quantum Phase Transition,” Communications Physics 6 (2023): 214, 10.1038/s42005-023-01332-8.

[101] G. C. Ménard , S. Guissart , C. Brun , et al., “Coherent Long‐Range Magnetic Bound States in a Superconductor,” Nature Physics 11 (2015): 1013–1016, 10.1038/nphys3508.

[102] S.‐H. Ji , T. Zhang , Y.‐S. Fu , et al., “High‐Resolution Scanning Tunneling Spectroscopy of Magnetic Impurity Induced Bound States in the Superconducting Gap of Pb Thin Films,” Physical Review Letters 100 (2008): 226801, 10.1103/PhysRevLett.100.226801. 18643441

[103] N. Hatter , B. W. Heinrich , M. Ruby , J. I. Pascual , and K. J. Franke , “Magnetic Anisotropy in Shiba Bound States Across a Quantum Phase Transition,” Nature Communications 6 (2015): 8988, 10.1038/ncomms9988. PMC4674822 26603561

[104] M. Ruby , Y. Peng , F. Von Oppen , B. W. Heinrich , and K. J. Franke , “Orbital Picture of Yu‐Shiba‐Rusinov Multiplets,” Physical Review Letters 117 (2016): 186801, 10.1103/PhysRevLett.117.186801. 27835014

[105] A. Yazdani , B. A. Jones , C. P. Lutz , M. F. Crommie , and D. M. Eigler , “Probing the Local Effects of Magnetic Impurities on Superconductivity,” Science 275 (1997): 1767–1770, 10.1126/science.275.5307.1767. 9065395

[106] S. Kezilebieke , R. Žitko , M. Dvorak , T. Ojanen , and P. Liljeroth , “Observation of Coexistence of Yu‐Shiba‐Rusinov States and Spin‐Flip Excitations,” Nano Letters 19 (2019): 4614–4619, 10.1021/acs.nanolett.9b01583. 31251066 PMC6628613

[107] S. M. F. Shahed , F. Ara , M. I. Hossain , K. Katoh , M. Yamashita , and T. Komeda , “Observation of Yu–Shiba–Rusinov States and Inelastic Tunneling Spectroscopy for Intramolecule Magnetic Exchange Interaction Energy of Terbium Phthalocyanine (TbPc) Species Adsorbed on Superconductor NbSe_2_ ,” ACS Nano 16 (2022): 7651–7661, 10.1021/acsnano.1c11221. 35467334 PMC9134493

[108] J. G. Rodrigo and S. Vieira , “STM Study of Multiband Superconductivity in NbSe_2_ Using a Superconducting Tip,” Physica C: Superconductivity 404 (2004): 306–310, 10.1016/j.physc.2003.10.030.

[109] M. E. Flatté and D. E. Reynolds , “Local Spectrum of a Superconductor as a Probe of Interactions Between Magnetic Impurities,” Physical Review B 61 (2000): 14810–14814, 10.1103/PhysRevB.61.14810.

[110] T. Meng , J. Klinovaja , S. Hoffman , P. Simon , and D. Loss , “Superconducting Gap Renormalization Around Two Magnetic Impurities: From Shiba to Andreev Bound States,” Physical Review B 92 (2015): 064503, 10.1103/PhysRevB.92.064503.

[111] J. Röntynen and T. Ojanen , “Topological Superconductivity and High Chern Numbers in 2D Ferromagnetic Shiba Lattices,” Physical Review Letters 114 (2015): 236803, 10.1103/PhysRevLett.114.236803. 26196820

[112] K. J. Franke , G. Schulze , and J. I. Pascual , “Competition of Superconducting Phenomena and Kondo Screening at the Nanoscale,” Science 332 (2011): 940–944, 10.1126/science.1202204. 21596987

[113] N. Hatter , B. W. Heinrich , D. Rolf , and K. J. Franke , “Scaling of Yu‐Shiba‐Rusinov Energies in the Weak‐Coupling Kondo Regime,” Nature Communications 8 (2017): 2016, 10.1038/s41467-017-02277-7. PMC5722882 29222411

[114] C. Rubio‐Verdú , J. Zaldívar , R. Žitko , and J. I. Pascual , “Coupled Yu‐Shiba‐Rusinov States Induced by a Many‐Body Molecular Spin on a Superconductor,” Physical Review Letters 126 (2021): 017001, 10.1103/PhysRevLett.126.017001. 33480757

[115] C. Li , V. Pokorný , M. Žonda , et al., “Individual Assembly of Radical Molecules on Superconductors: Demonstrating Quantum Spin Behavior and Bistable Charge Rearrangement,” ACS Nano 19 (2025): 3403–3413, 10.1021/acsnano.4c12387. 39806870 PMC11781030

[116] F. von Oppen and K. J. Franke , “Yu‐Shiba‐Rusinov States in Real Metals,” Physical Review B 103 (2021): 205424, 10.1103/PhysRevB.103.205424.

[117] J. Kügel , P.‐J. Hsu , M. Böhme , et al., “Jahn–Teller Splitting in Single Adsorbed Molecules Revealed by Isospin‐Flip Excitations,” Physical Review Letters 121 (2018): 226402, 10.1103/PhysRevLett.121.226402. 30547609

[118] J. Kügel , M. Karolak , J. Senkpiel , P.‐J. Hsu , G. Sangiovanni , and M. Bode , “Relevance of Hybridization and Filling of 3d Orbitals for the Kondo Effect in Transition Metal Phthalocyanines,” Nano Letters 14 (2014): 3895–3902, 10.1021/nl501150k. 24871813

[119] Y. Xing , H. Chen , B. Hu , Y. Ye , W. A. Hofer , and H.‐J. Gao , “Reversible Switching of Kondo Resonance in a Single‐Molecule Junction,” Nano Research 15 (2022): 1466–1471, 10.1007/s12274-021-3688-1.

[120] J. Shuai‐Hua , F. Ying‐Shuang , Z. Tong , et al., “Kondo Effect in Self‐Assembled Manganese Phthalocyanine Monolayer on Pb Islands,” Chinese Physics Letters 27 (2010): 087202, 10.1088/0256-307X/27/8/087202.

[121] Y.‐S. Fu , S.‐H. Ji , X. Chen , et al., “Manipulating the Kondo Resonance Through Quantum Size Effects,” Physical Review Letters 99 (2007): 256601, 10.1103/PhysRevLett.99.256601. 18233541

[122] T. Matsuura , “The Effects of Impurities on Superconductors With Kondo Effect,” Progress of Theoretical Physics 57 (1977): 1823–1835, 10.1143/PTP.57.1823.

[123] P. Berggren and J. Fransson , “Spin Inelastic Electron Tunneling Spectroscopy on Local Magnetic Moment Embedded in Josephson Junction,” EPL 108 (2014): 67009, 10.1209/0295-5075/108/67009.

[124] P. Berggren and J. Fransson , “Theory of Spin Inelastic Tunneling Spectroscopy for Superconductor‐Superconductor and Superconductor‐Metal Junctions,” Physical Review B 91 (2015): 205438, 10.1103/PhysRevB.91.205438.

[125] K. G. Wilson , “The Renormalization Group: Critical Phenomena and the Kondo Problem,” Reviews of Modern Physics 47 (1975): 773–840, 10.1103/RevModPhys.47.773.

[126] J. Kügel , M. Karolak , A. Krönlein , et al., “State Identification and Tunable Kondo Effect of MnPc on Ag(001),” Physical Review B 91 (2015): 235130, 10.1103/PhysRevB.91.235130.

[127] A. Mugarza , C. Krull , R. Robles , S. Stepanow , G. Ceballos , and P. Gambardella , “Spin Coupling and Relaxation inside Molecule–Metal Contacts,” Nature Communications 2 (2011): 490, 10.1038/ncomms1497. 21971505

[128] C. Daniel and C. Gourlaouen , “Chemical Bonding Alteration Upon Electronic Excitation in Transition Metal Complexes,” Coordination Chemistry Reviews 344 (2017): 131–149, 10.1016/j.ccr.2016.10.010.

[129] M. Baljozović , J. Pijeat , S. Campidelli , and K.‐H. Ernst , “Planar and Curved π‐Extended Porphyrins by on‐Surface Cyclodehydrogenation,” Journal of the American Chemical Society 146 (2024): 34600–34608, 10.1021/jacs.4c12460. 39629975 PMC11664915

[130] Y. Zhao , K. Jiang , C. Li , et al., “Quantum Nanomagnets in on‐Surface Metal‐Free Porphyrin Chains,” Nature Chemistry 15 (2023): 53–60, 10.1038/s41557-022-01061-5. 36280765

[131] D. B. Rice , D. Wong , T. Weyhermüller , F. Neese , and S. DeBeer , “The Spin‐Forbidden Transition in Iron(IV)‐Oxo Catalysts Relevant to Two‐State Reactivity,” Science Advances 10 (2024): ado1603, 10.1126/sciadv.ado1603. PMC11212722 38941457

[132] R. Li , N. Li , H. Wang , et al., “Tuning the Spin‐Related Transport Properties of FePc on Au(111) Through Single‐Molecule Chemistry,” Chemical Communications 54 (2018): 9135–9138, 10.1039/C8CC02994F. 30059079

[133] Y. H. Chang , H. Kim , S.‐J. Kahng , and Y.‐H. Kim , “Axial Coordination and Electronic Structure of Diatomic NO, CO, and O_2_ Molecules Adsorbed Onto Co‐Tetraphenylporphyrin on Au(111), Ag(111), and Cu(111): A Density‐Functional Theory Study,” Dalton Transactions 45 (2016): 16673–16681, 10.1039/C6DT01965J. 27711671

[134] W. Hu , D. Wang , Q. Ma , B. J. Reinhart , X. Zhang , and J. Huang , “The Impact of Axial Ligation on the Excited State Dynamics of Cobalt(II) Phthalocyanine,” Journal of Photochemistry and Photobiology 11 (2022): 100132, 10.1016/j.jpap.2022.100132.

[135] A. Stróżecka , M. Soriano , J. I. Pascual , and J. J. Palacios , “Reversible Change of the Spin State in a Manganese Phthalocyanine by Coordination of CO Molecule,” Physical Review Letters 109 (2012): 147202, 10.1103/PhysRevLett.109.147202. 23083274

[136] W. Huang , P. Greule , M. Stark , et al., “Probing Magnetism in Self‐Assembled Organometallic Complexes Using Kondo Spectroscopy,” ACS Nano 19 (2025): 1190–1197, 10.1021/acsnano.4c13172. 39757545

[137] C. Isvoranu , B. Wang , E. Ataman , et al., “Comparison of the Carbonyl and Nitrosyl Complexes Formed by Adsorption of CO and NO on Monolayers of Iron Phthalocyanine on Au(111),” Journal of Physical Chemistry C 115 (2011): 24718–24727, 10.1021/jp204461k.

[138] K. Flechtner , A. Kretschmann , H.‐P. Steinrück , and J. M. Gottfried , “NO‐Induced Reversible Switching of the Electronic Interaction Between a Porphyrin‐Coordinated Cobalt Ion and a Silver Surface,” Journal of the American Chemical Society 129 (2007): 12110–12111, 10.1021/ja0756725. 17877358

[139] W. Hieringer , K. Flechtner , A. Kretschmann , et al., “The Surface Trans Effect: Influence of Axial Ligands on the Surface Chemical Bonds of Adsorbed Metalloporphyrins,” Journal of the American Chemical Society 133 (2011): 6206–6222, 10.1021/ja1093502. 21462965

[140] A. B. Gaspar , V. Ksenofontov , M. Seredyuk , and P. Gütlich , “Multifunctionality in Spin Crossover Materials,” Coordination Chemistry Reviews 249 (2005): 2661–2676, 10.1016/j.ccr.2005.04.028.

[141] X. Liu , H. Yang , H. Harb , et al., “Shape‐Persistent Ladder Molecules Exhibit Nanogap‐Independent Conductance in Single‐Molecule Junctions,” Nature Chemistry 16 (2024): 1772–1780, 10.1038/s41557-024-01619-5. 39187723

[142] K. R. Parenti , R. Chesler , G. He , et al., “Quantum Interference Effects Elucidate Triplet‐Pair Formation Dynamics in Intramolecular Singlet‐Fission Molecules,” Nature Chemistry 15 (2023): 339–346, 10.1038/s41557-022-01107-8. 36585444

[143] M. O. Hight , J. Y. Wong , A. E. Pimentel , and T. A. Su , “Intramolecular London Dispersion Interactions in Single‐Molecule Junctions,” Journal of the American Chemical Society 146 (2024): 4716–4726, 10.1021/jacs.3c12183. 38325000 PMC10885141

[144] J. J. Parks , A. R. Champagne , T. A. Costi , et al., “Mechanical Control of Spin States in Spin‐1 Molecules and the Underscreened Kondo Effect,” Science 328 (2010): 1370–1373, 10.1126/science.1186874. 20538943

[145] B. R. Mullaney , L. Goux‐Capes , D. J. Price , G. Chastanet , J.‐F. Létard , and C. J. Kepert , “Spin Crossover‐Induced Colossal Positive and Negative Thermal Expansion in a Nanoporous Coordination Framework Material,” Nature Communications 8 (2017): 1053, 10.1038/s41467-017-00776-1. PMC5648752 29051479

[146] M. M. Paquette , D. Plaul , A. Kurimoto , B. O. Patrick , and N. L. Frank , “Opto‐Spintronics: Photoisomerization‐Induced Spin State Switching at 300 K in Photochrome Cobalt–Dioxolene Thin Films,” Journal of the American Chemical Society 140 (2018): 14990–15000, 10.1021/jacs.8b09190. 30351017

[147] T. Matsumoto , G. N. Newton , T. Shiga , et al., “Programmable Spin‐State Switching in a Mixed‐Valence Spin‐Crossover Iron Grid,” Nature Communications 5 (2014): 3865, 10.1038/ncomms4865. 24832549

[148] M. Bernien , D. Wiedemann , C. F. Hermanns , et al., “Spin Crossover in a Vacuum‐Deposited Submonolayer of a Molecular Iron(II) Complex,” Journal of Physical Chemistry Letters 3 (2012): 3431–3434, 10.1021/jz3011805. 26290968

[149] R. Hiraoka , E. Minamitani , R. Arafune , et al., “Single‐Molecule Quantum Dot as a Kondo Simulator,” Nature Communications 8 (2017): 16012, 10.1038/ncomms16012. PMC5497065 28665404

[150] A. F. Otte , M. Ternes , K. von Bergmann , et al., “The Role of Magnetic Anisotropy in the Kondo Effect,” Nature Physics 4 (2008): 847–850, 10.1038/nphys1072.

[151] J. C. Oberg , M. R. Calvo , F. Delgado , et al., “Control of Single‐Spin Magnetic Anisotropy by Exchange Coupling,” Nature Nanotechnology 9 (2014): 64–68, 10.1038/nnano.2013.264. 24317285

[152] Q. Chen , H. Du , X. Meng , et al., “Microscopic Mechanism of Coexisting Electron Spin Resonance and Kondo Resonance in a Single Iron Phthalocyanine Molecule,” Physical Review Letters 135 (2025): 086403, 10.1103/cgq3-dyxb. 40929327

[153] W. Huang , K. H. Au‐Yeung , P. Greule , et al., “An Electrically Controlled Single‐Molecule Spin Switch,” Nature Communications 16 (2025): 8242, 10.1038/s41467-025-63574-0. PMC12417546 40921783

[154] J. Girovsky , J. Nowakowski , M. E. Ali , et al., “Long‐Range Ferrimagnetic Order in a Two‐Dimensional Supramolecular Kondo Lattice,” Nature Communications 8 (2017): 15388, 10.1038/ncomms15388. PMC5458152 28530247

[155] Q. Zhang , W.‐Y. He , Y. Zhang , et al., “Quantum Spin Liquid Signatures in Monolayer 1T‐NbSe_2_ ,” Nature Communications 15 (2024): 2336, 10.1038/s41467-024-46612-1. PMC10940636 38485980

[156] S. Chakraborty , G. Fratesi , and A. Ravikumar , “Defect Controlled Spin state Transitions in FePc Adsorbed CrI_3_ ,” Surfaces and Interfaces 50 (2024): 104452, 10.1016/j.surfin.2024.104452.

[157] C. Bacaksiz and M. Fyta , “Phthalocyanine Adsorbed on Monolayer CrI_3_: Tailoring Their Magnetic Properties,” ACS Omega 9 (2024): 34589–34596, 10.1021/acsomega.4c02708. 39157117 PMC11325395

[158] P. Willke , T. Bilgeri , X. Zhang , et al., “Coherent Spin Control of Single Molecules on a Surface,” ACS Nano 15 (2021): 17959–17965, 10.1021/acsnano.1c06394. 34767351

[159] X. Zhang , C. Wolf , Y. Wang , et al., “Electron Spin Resonance of Single Iron Phthalocyanine Molecules and Role of Their Non‐Localized Spins in Magnetic Interactions,” Nature Chemistry 14 (2022): 59–65, 10.1038/s41557-021-00827-7. 34764471

[160] G. Czap , P. J. Wagner , F. Xue , et al., “Probing and Imaging Spin Interactions With a Magnetic Single‐Molecule Sensor,” Science 364 (2019): 670–673, 10.1126/science.aaw7505. 31097665

[161] C. Zhang , F. Zhou , H. Jin , et al., “Tailored Spin Coupling of Single‐Molecule Magnets With a Single Charge‐Density‐Wave Metal Layer,” Journal of the American Chemical Society 148 (2026): 11953–11961, 10.1021/jacs.5c21952. 41817978 PMC13022852

